# Amniotes co-opt intrinsic genetic instability to protect germ-line genome integrity

**DOI:** 10.1038/s41467-023-36354-x

**Published:** 2023-02-13

**Authors:** Yu H. Sun, Hongxiao Cui, Chi Song, Jiafei Teng Shen, Xiaoyu Zhuo, Ruoqiao Huiyi Wang, Xiaohui Yu, Rudo Ndamba, Qian Mu, Hanwen Gu, Duolin Wang, Gayathri Guru Murthy, Pidong Li, Fan Liang, Lei Liu, Qing Tao, Ying Wang, Sara Orlowski, Qi Xu, Huaijun Zhou, Jarra Jagne, Omer Gokcumen, Nick Anthony, Xin Zhao, Xin Zhiguo Li

**Affiliations:** 1 Center for RNA Biology: From Genome to Therapeutics, Department of Biochemistry and Biophysics, University of Rochester Medical Center, Rochester, NY 14642 USA; 2 College of Animal Science and Technology, Northwest A&F University, Yangling, Shaanxi 712100 China; 3 College of Public Health, Division of Biostatistics, The Ohio State University, Columbus, OH 43210 USA; 4 International Institutes of Medicine, The Fourth Affiliated Hospital, Zhejiang University School of Medicine, Yiwu, Zhejiang 322000 China; 5 Department of Genetics, The Edison Family Center for Genome Sciences and Systems Biology, Washington University School of Medicine, St. Louis, MO 63110 USA; 6 Grandomics Biosciences Co., Ltd, Beijing, 102206 China; 7 Department of Animal Science, University of California, Davis, CA 95616 USA; 8 Department of Poultry Science, University of Arkansas, Fayetteville, AR 72701 USA; 9 Department of Animal Science, McGill University, Quebec, H9X 3V9 Canada; 10 Animal Health Diagnostic Center, Cornell University College of Veterinary Medicine, Ithaca, NY 14850 USA; 11 Department of Biological Sciences, University at Buffalo, State University of New York, Buffalo, NY 14260 USA

**Keywords:** Genome informatics, Evolutionary genetics, Piwi RNAs, DNA transposable elements

## Abstract

Unlike PIWI-interacting RNA (piRNA) in other species that mostly target transposable elements (TEs), >80% of piRNAs in adult mammalian testes lack obvious targets. However, mammalian piRNA sequences and piRNA-producing loci evolve more rapidly than the rest of the genome for unknown reasons. Here, through comparative studies of chickens, ducks, mice, and humans, as well as long-read nanopore sequencing on diverse chicken breeds, we find that piRNA loci across amniotes experience: (1) a high local mutation rate of structural variations (SVs, mutations ≥ 50 bp in size); (2) positive selection to suppress young and actively mobilizing TEs commencing at the pachytene stage of meiosis during germ cell development; and (3) negative selection to purge deleterious SV hotspots. Our results indicate that genetic instability at pachytene piRNA loci, while producing certain pathogenic SVs, also protects genome integrity against TE mobilization by driving the formation of rapid-evolving piRNA sequences.

## Abstract (web summary)

Pachytene Piwi-interacting RNAs (piRNAs) expressed in mammalian germ lines are abundant, but their evolution and function are not fully understood. Here, the authors find that pachytene piRNA loci are hotspots of structural variation, which underlies rapid piRNA birth, divergence, and loss.

## Introduction

PIWI-interacting RNAs (piRNAs) are essential for animal fertility. They are 24–35 nt long, have 2´-*O*-methyl-modified 3´ termini, and associate with PIWI proteins, a specialized family of Argonaute proteins expressed in germ cells. A conserved function of piRNAs across all bilateral animals is to silence sequence-complementary transposable elements (TEs)^1–4^. Adult mammals express high levels of a unique class of piRNAs that evolve at an exceptionally rapid rate. Two features distinguish adult mammalian piRNAs, also known as pachytene piRNAs, from either TE-rich primitive piRNAs found in fruit flies and zebrafish or pre-pachytene piRNAs in mammals: (1) pachytene piRNAs are expressed during the pachytene stage of meiosis; and (2) they are derived from intergenic regions where TEs are not dominant. While most pachytene piRNAs lack obvious targets, neither the copy numbers nor nucleotide sequences of pachytene piRNA loci are conserved^5–7^, and many of them are not found in syntenic regions even in closely related mammals^8,9^. Pachytene piRNAs have been proposed to either regulate mRNAs^10–14^ or stabilize PIWI proteins for a function that does not require piRNA-guided sequence specificity^15^. Such proposed functions are difficult to reconcile with pachytene piRNAs’ rapid evolution, and this rapid evolution and their redundant distribution across multiple loci on the genome also complicates their functional study. Therefore, it is still unclear what function pachytene piRNAs have and what promotes their rapid divergence.

## Results

### Avian pachytene piRNAs diverge rapidly

**Fig. 1:**
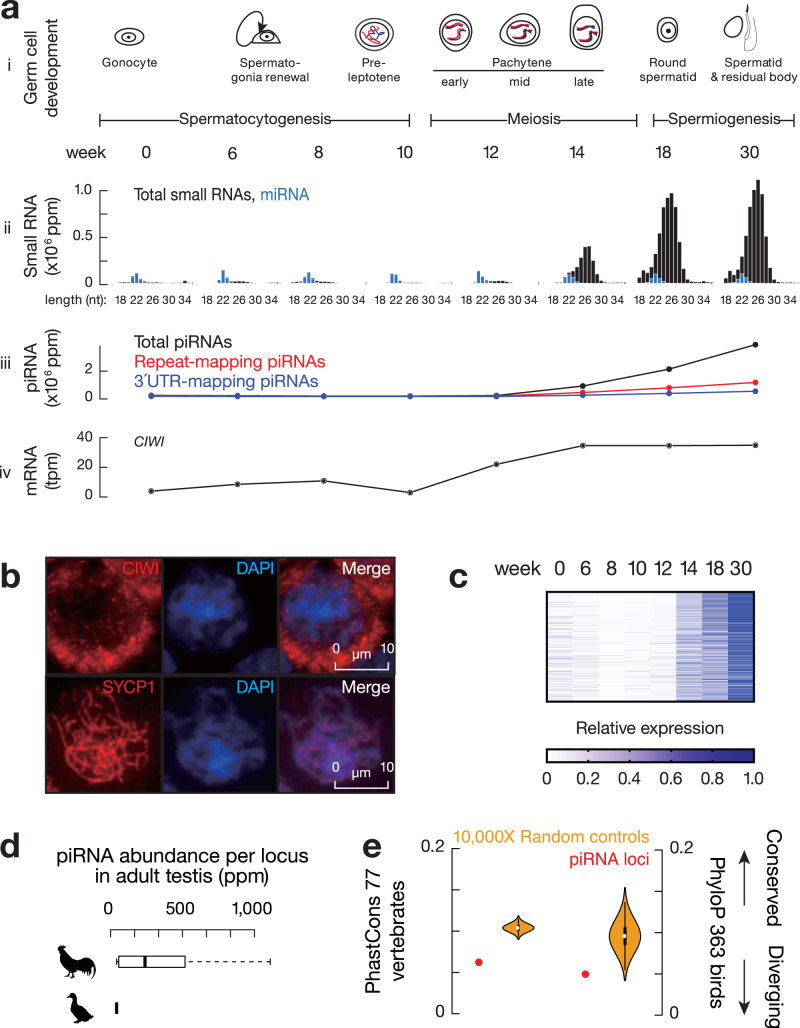
Existence of pachytene piRNAs in chickens. **a** Roosters express pachytene piRNAs during spermatogenesis. (i) Key biological events during chicken spermatogenesis. (ii) Length distribution of total small RNAs. Ppm, parts per million. Blue, miRNAs. (iii) Abundance of piRNAs as measured by small RNA-seq. (iv) Expression of *CIWI* as measured by RNA-seq. Tpm, transcript per million. **b** Immunolabeling of squashed pachytene spermatocytes from adult chicken testes using anti-CIWI, anti-SYCP1, and DAPI. Scale bar, 10 µm. SYCP1, marker for synaptonemal complex formed during pachynema. We took at least 30 pictures and the representative pictures were shown. **c** Heatmap of normalized piRNA abundance per piRNA locus across the eight developmental stages of chicken testes. **d** Box plots of piRNA abundance at piRNA loci (*n* = 1321) in adult chicken testes and at their homolog regions (*n* = 637) in adult duck testes. Ppm: parts per million. Box plots show the 25th and 75th percentiles, whiskers represent the 5th and 95th percentiles, and midlines show median values. **e** Median value of (left) the mean phastCons score from 77 vertebrate genome alignments (probability that each nucleotide belongs to a conserved element) and (right) the mean phyloP score from 363 bird genome alignments (represent −log *p-*values under a null hypothesis of neutral evolution) of piRNA loci (red, *n* = 1321) and randomly shuffled control sequences (yellow, *n* = 10,000). Violin plots represent the medians of randomly shuffled control sequences that were computed 10,000 times.

To understand whether pachytene piRNAs are specific to mammals, we looked for their presence in *Gallus gallus* (chickens), which diverged from mammals 330 million years ago^16^. We have previously detected predominantly non-TE piRNAs in adult chicken testes^17^, however, it was unclear whether these piRNAs were expressed during the pachytene stage of meiosis. To characterize the dynamics of the chicken piRNA repertoire, we analyzed the first wave of spermatogenesis (Fig. 1a, i and Supplementary Fig. 1a) by collecting chicken testes at eight key developmental stages (day 1 to 30 weeks—sexual maturity; Fig. 1a, i) from a broiler breeder breed (Athens Canadian Random Bred, ACRB^18^). The majority of piRNAs were expressed during the transition from 12 to 18 weeks (Fig. 1a, ii and iii and Supplementary Fig. 1b), the period when meiosis occurs during the first wave of spermatogenesis (Supplementary Fig. 1a). This stage coincides with the mRNA expression of *CIWI*, a PIWI gene whose ortholog in mice specifically binds to pachytene piRNAs^17^ (Fig. 1a, iv). We also detected stage-specific staining of CIWI protein in the cytosol of pachytene spermatocytes (Fig. 1b and Supplementary Fig. 1c). Plotting piRNA abundance at each piRNA locus during the eight developmental stages, we detected a burst of expression at the pachytene stage with little piRNA existing in prior stages (Fig. 1c), indicating that most, if not all, piRNAs in adult testes are pachytene piRNAs. Similar to mammalian pachytene piRNAs, most of the piRNAs from adult chicken testes were not derived from repetitive regions nor genic regions (Fig. 1a, iii). These results demonstrate the existence of pachytene piRNAs in chickens, suggesting a function of pachytene piRNAs during germ cell development shared by birds and mammals.

To test for conservation of chicken pachytene piRNA loci in closely related bird species, we searched for their homologs in *Anas platyrhynchos domesticus* (Pekin duck), which diverged from chickens approximately 90–100 million years ago^19^. Overall, 39% of the chicken genome has homologous sequences in the duck genome detected by DNA in-situ hybridization^20^. However, we were able to identify homologs for only ~10% (136 out of 1321) of chicken piRNA loci in the duck genome, indicating an absence of homologous sequences of most chicken piRNA loci. At the functional level, while we detected abundant piRNAs in duck testes as demonstrated by a characteristic length distribution and resistance to oxidation due to their 2´-*O*-methyl-modified 3´ termini (Supplementary Fig. 1d), the 136 loci homologous to chicken piRNA loci no longer produce piRNAs (Fig. 1d). This comparison with ducks indicates that chicken piRNA loci undergo rapid gain and loss at both the sequence level (whether its homolog region exists) and the functional level (whether the homolog region produces piRNAs). Consistent with the lack of genome alignment at piRNA loci between chickens and ducks, compared to the exons and introns of both mRNAs and long non-coding RNAs (lncRNAs) as well as a set of randomly shuffled controls, piRNA loci displayed the lowest conservation scores among vertebrates (Supplementary Fig. 2a, piRNA loci vs. mRNA or lncRNA genes, two-tailed Wilcoxon signed rank test, *p <* 2.2 × 10^−16^; Fig. 1e, left, piRNA loci vs. a set of control sequences by randomly shuffling piRNA loci on the same chromosome, one-tailed permutation test, *p <* 1.0 × 10^−4^) and within birds (Fig. 1e, right, piRNA loci vs. a set of control sequences by randomly shuffling piRNA loci on the same chromosome, one-tailed permutation test, *p =* 4.0 × 10^−4^). Together with previous work in mammals^9,21^, we conclude that rapid divergence is a common feature for pachytene piRNAs in both mammals and birds. Considering that avian genomes display a high degree of evolutionary stasis in nucleotide sequence, gene synteny, and chromosomal structure compared to mammalian genomes^22^, the shared feature of rapid divergence across mammals and birds suggests unifying principles driving pachytene piRNA evolution.

### piRNA loci are SV hotspots in birds and mammals

**Fig. 2:**
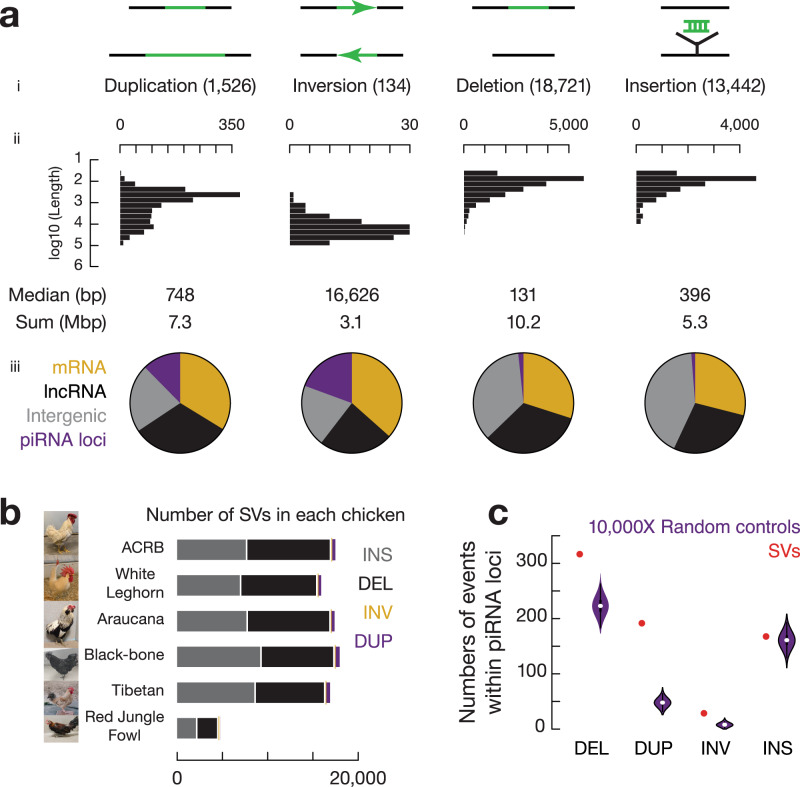
Chicken piRNA loci are SV hotspots. **a** The landscape of SVs in chickens. (i) Quantity of each type of SV, (ii) density plots showing their length distributions, and (iii) pie chart showing their overlapping genomic regions. **b** Bar plots of the quantity of SVs in each chicken. INS, insertion; DEL, deletion; INV, inversion; DUP, tandem duplication. The Red Jungle Fowl had a significantly lower number of SVs compared to that of domesticated chickens (Z score = −15.9). Given our Red Jungle Fowl is from the same population selected for reference genome sequencing, the 4934 SVs detected in Red Jungle Fowl likely underrepresented the level of genetic diversity in the wild chicken population. **c** The number of SVs (red) and randomly shuffled control sequences (purple) falling into the piRNA loci (*n* = 1321). Violin plots represent the randomly shuffled control sequences that were computed 10,000 times.

We decided to analyze the mutational events at pachytene piRNA loci over short evolutionary timescales using chickens as a model because the chicken genome is one-third the size of the human genome and includes a smaller fraction of TEs (10% vs 50%, respectively)^23^, which are less repetitive and therefore more tractable to work with both bioinformatically and experimentally. We sequenced six chickens from diverse breeds with distinct geographic distributions and specific traits (Supplementary Fig. 2b). To capture structural variations (SVs, mutations affecting ≥ 50 bp) with high resolution and fidelity^24,25^, we used Oxford Nanopore Technologies (ONT) long-read sequencing and achieved a depth of > 31× coverage per chicken, an average ONT-read length of 17 ± 6 kb, an average mappability of 95 ± 1%, an error rate of 14 ± 2% (Supplementary Data 1), and a total of 17,321 ± 777 SV events per domestic chicken compared to the reference genome from undomesticated wild chickens (Fig. 2a, b and Supplementary Fig. 2c). Pachytene piRNA loci constitute 0.98% of the chicken genome^26^, but larger frequencies of SVs occur at pachytene piRNA loci: 12.4% of tandem duplications (189 out of 1526), 19.4% of inversions (26 out of 134), 1.7% of deletions (314 out of 18,721), and 1.2% of insertions (165 out of 13,442) overlapped with piRNA loci (Fig. 2a, iii). We found that the enrichment of tandem duplications, inversions, and deletions (SVs in piRNA loci vs. a set of control sequences by randomly shuffling SVs on the same chromosome that fall into piRNA loci, one-tailed permutation test, *p <* 1.0 × 10^−4^, Fig. 2c), but not insertions (*p =* 0.38), were significant in piRNA loci. Such enrichments of SVs were not seen at the lncRNA genes (Supplementary Fig. 2d). We defined 192 SV hotspots, which account for 1.1% of the chicken genome and include 7.7% of SVs. Chicken pachytene piRNA loci are significantly overlapped with these SV hotspots (Pachytene piRNA loci overlapping with SV hotspots vs. a set of control sequences by randomly shuffling piRNA loci on the same chromosome that overlap with SV hotspots, one-tailed permutation test, *p =* 3.0 × 10^−4^, Supplementary Fig. 2d). Thus, with non-random distribution of SVs in the chicken genome, chicken piRNA loci represent SV hotspots.

**Fig. 3:**
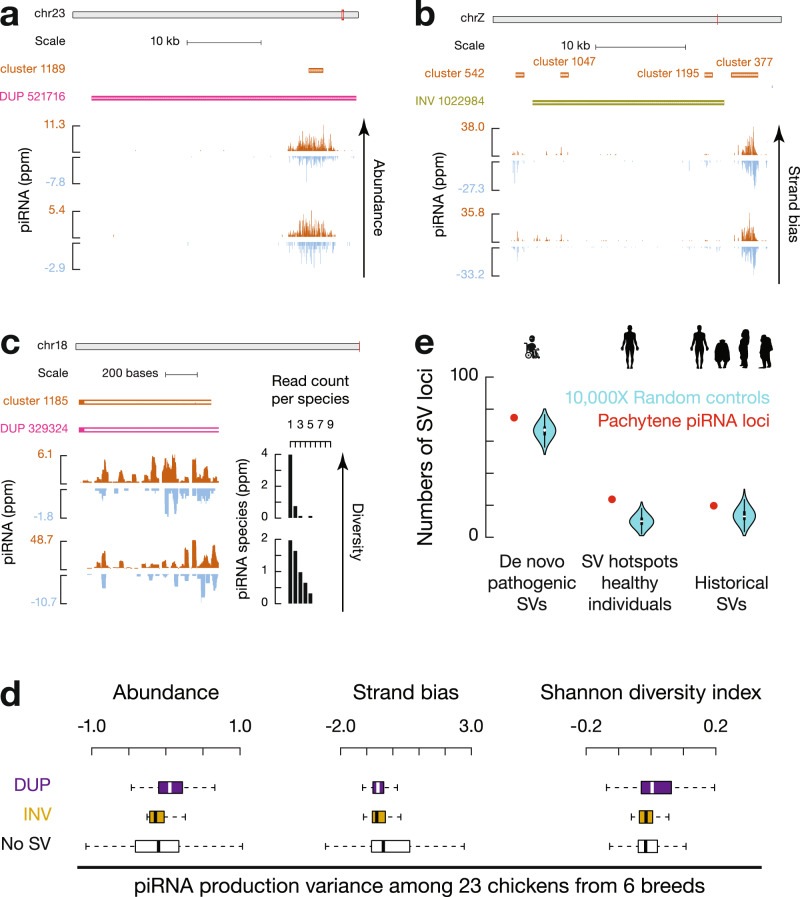
Conserved mechanisms to achieve piRNA plasticity. **a** Example of a duplication overlapping with a piRNA locus and its piRNA abundance from two chicken individuals. Blue represents Watson strand mapping reads; Red represents Crick strand mapping reads. Ppm, parts per million. **b** Example of an inversion overlapping with two piRNA loci (cluster 1047 and cluster 1195) along with their nonoverlapping control piRNA loci (cluster 542 and cluster 377) and their piRNA abundance from two chicken individuals. Blue represents Watson strand mapping reads; Red represents Crick strand mapping reads. Ppm, parts per million. **c** Example of a duplication overlapping with a piRNA locus. (Left) piRNA abundance from two chicken individuals. Blue represents Watson strand mapping reads; Red represents Crick strand mapping reads. Ppm, parts per million. (Right) piRNA species abundance from the two chicken individuals that have read counts of 1 to 9. **d** Box plots of piRNA variance of Abundance (left), Strand bias (middle), and Shannon diversity index (right) among 23 chickens from 6 breeds. Box plots show the 25th and 75th percentiles, whiskers represent the 5th and 95th percentiles, and midlines show median values. **e** Number of human pachytene piRNA loci (red) and randomly shuffled control sequences (aquamarine) overlapping with SV hotspots within de novo pathogenic SVs detected in patients (left), healthy human populations (middle), and historical SVs in the common ancestor of humans and great apes (right). Violin plots represent the medians of randomly shuffled control sequences that were computed 10,000 times.

To test the impact of SVs at piRNA loci on piRNA polymorphisms, we sequenced small RNAs individually in a pool of chickens from the six chicken breeds (3–5 biological replicates for each breed). Although insertions are not significantly enriched at piRNA loci compared to bulk genomes (Fig. 2a, c), we found that the 165 novel insertions into piRNA loci add new sequences to piRNA pools (Supplementary Fig. 3a, b). SVs in piRNA loci are also associated with changes of piRNAs in expression dosage (Fig. 3a), sense/anti-sense orientation (Fig. 3b), and relative abundance of different piRNA species (Fig. 3c). We quantified the individual variance of piRNA abundance, strand bias, and Shannon diversity index measurements and found that SV regions within piRNA loci displayed significantly higher variance than piRNA loci lacking SVs (SV regions vs. loci without SVs, two-tailed Wilcoxon signed rank test, *p ≤* 3.0 × 10^−8^, Fig. 3d, Supplementary Fig. 3c). Therefore, overlapping with SV hotspots correlates with the rapid divergence of piRNAs.

We next asked whether the association between SV hotspots and piRNA loci was avian-specific or common to all amniotes. To distinguish these two possibilities, we analyzed the 278 SV hotspots recently discovered using long-read sequencing from 35 healthy human individuals from 25 human populations^27^. Pachytene piRNA loci (Supplementary Fig. 3d, right, SV loci vs. a set of control sequences by randomly shuffling SV loci on the same chromosome, one-tailed permutation test, *p* = 1.5 × 10^−3^), but not pre-pachytene piRNA loci (Supplementary Fig. 3d, left, *p* = 0.16), significantly overlapped with human SV hotspots (Fig. 3e, middle, piRNA loci vs. a set of control sequences by randomly shuffling piRNA loci on the same chromosome, one-tailed permutation test, *p* < 1.0 × 10^−4^). The high mutation rate of SVs at human pachytene piRNA loci explains previously reported low conservation scores and increased copy number variations among mammals^7,28^, indicating that, like chicken piRNA loci, human pachytene piRNA loci are also SV hotspots. Thus, the high local mutation rate of SVs serves as a conserved force contributing to the rapid divergence of pachytene piRNAs across amniotes.

### Convergent evolution of piRNA loci overlapping with SV hotspots

**Fig. 4:**
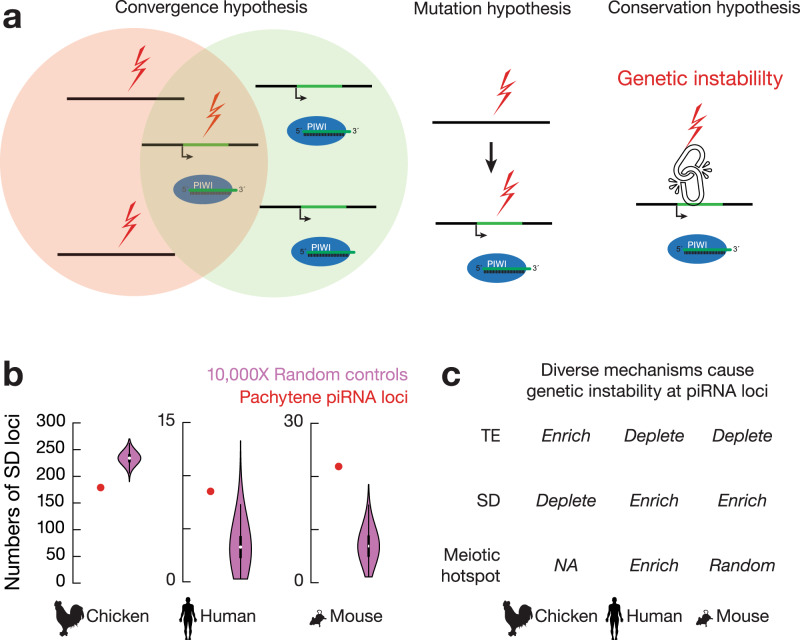
Convergent evolution drives the association between SV hotspots and pachytene piRNA loci. **a** Three models explain the association between pachytene piRNA loci and SV hotspots. **b** Number of pachytene piRNA loci (red) and randomly shuffled control sequences (magenta) overlapping with SDs from chickens (*n* = 861), humans (*n* = 3802), and mice (*n* = 659,775). Violin plots represent the medians of randomly shuffled control sequences that were computed 10,000 times. **c** Summary of diverse mutational mechanisms contributing to genetic instability at pachytene piRNA loci.

We envision three possible mechanisms resulting in the association between piRNA loci and SV hotspots (Fig. 4a): (1) piRNA loci and SV hotspots originated independently, and their overlap is adaptively selected for through convergent evolution under common selective pressures (convergence hypothesis); (2) SV hotspots appear first and increase the chance of genomic regions to evolve into piRNA loci (mutation hypothesis); and (3) conserved molecular machinery links piRNA biogenesis to SV formation (conservation hypothesis), with either the production of piRNAs leading to genetic instability or the DNA damage of SVs triggering piRNA production. The “convergence” and “conservation” hypotheses predict that ancient piRNA loci should harbor more mutations than recent piRNA loci, while the “mutation” hypothesis predicts that all these piRNA loci should carry similar mutation levels because both recent piRNA loci and ancient piRNA loci arose from existing SV hotspots. Among the 88 human pachytene piRNA loci, the 29 loci shared with other eutherians carry more polymorphisms than the 43 primate-only piRNA loci and 16 human-specific piRNA loci^7^. Furthermore, only the human-specific piRNA loci significantly overlapped with SV hotspots (Supplementary Fig. 4a, left, piRNA loci vs. a set of control sequences by randomly shuffling piRNA loci on the same chromosome, one-tailed permutation test, *p* < 1.0 × 10^−4^), probably due to the selection to eliminate SVs over evolutionary time. Altogether, our data suggest that the association between piRNA loci and hotspots has survived long-term selection, and with young and old piRNA loci carrying different mutation levels, our data rule out the “mutation” hypothesis.

There is no general association between SV hotspots and piRNA loci, as only a subset of SV hotspots produces piRNAs in chickens and humans (Supplementary Fig. 4b), which is inconsistent with the “conservation” hypothesis and suggests that no piRNA biogenic machinery recognizes SVs. To test the possibility that the generation of piRNAs makes their production loci unstable, we analyzed 3´UTR piRNAs, a class of piRNAs that derive from a subset of protein-coding mRNAs and function in fine-tuning protein levels rather than silencing active TEs^29^. This class of piRNAs shares the same biogenic mechanisms as piRNAs derived from lncRNAs encoded from piRNA loci^29^. We found that chicken SVs are not significantly increased in these genic piRNA regions (Supplementary Fig. 4c, SV loci vs. a set of control sequences by randomly shuffling SV loci on the same chromosome, one-tailed permutation test, *p* ≥ 0.34), nor are they significantly increased compared to SVs falling into all protein-coding genes (χ^2^, *p* = 0.40). Ruling out the possibility that negative selection eliminates the outcome of SVs in protein-coding regions and only keeps the outcomes with efficient repair despite increased genetic instability, these genic piRNA regions do not exhibit increased nucleotide divergence with a similar high conservation score as other protein coding genes (Supplementary Fig. 2a, genic piRNA vs. mRNA gene, two-tailed Wilcoxon signed rank test, *p* ≥ 0.46). Thus, neither piRNA production itself increases genetic instability nor do SVs trigger piRNA production. Therefore, our data rule out the “conservation” hypothesis.

To test whether SV hotspots originate independently, we traced the mutational mechanisms of piRNA loci across species. Considering that TEs and segmental duplications (SDs, over 1 kb size, > 90% identity) are prone to form SVs^30–32^, we found that chicken piRNA loci were significantly enriched for TEs compared to bulk sequences (Supplementary Fig. 4d, piRNA loci vs. a set of control sequences by randomly shuffling piRNA loci on the same chromosome, one-tailed permutation test, *p* < 1.0 × 10^−4^) but were depleted for SDs (Fig. 4b, left, piRNA loci *vs.* a set of control sequences by randomly shuffling piRNA loci on the same chromosome, one-tailed permutation test, *p* < 1.0 × 10^−4^). In contrast, while human pachytene piRNA loci are relatively depleted of TEs^7,33^, they were significantly enriched with SDs^34^ (Fig. 4b, middle, piRNA loci vs. a set of control sequences by randomly shuffling piRNA loci on the same chromosome, one-tailed permutation test, *p* = 8.5 × 10^−3^). Although SDs in humans and mice have distinct distributions^35^, mouse pachytene piRNA loci are also significantly enriched for SDs (Fig. 4b, right, piRNA loci vs. a set of control sequences by randomly shuffling piRNA loci on the same chromosome, one-tailed permutation test, *p* < 1.0 × 10^−4^). Furthermore, we found that human pachytene piRNA loci (Supplementary Fig. 4e, piRNA loci vs. a set of control sequences by randomly shuffling piRNA loci on the same chromosome, one-tailed permutation test, *p* = 1.3 × 10^−3^), but not mice pachytene piRNA loci (Supplementary Fig. 4e, piRNA loci vs. a set of control sequences by randomly shuffling piRNA loci on the same chromosome, one-tailed permutation test, *p* = 0.28), are significantly overlapped with meiotic double-strand break (DSB) hotspots. We also rule out the possibility that TE transposition activity contributes to instability at piRNA loci by demonstrating the low number of novel TE insertions at piRNA loci and the considerable distance between novel TE integration sites and piRNA loci in the chicken genome (Supplementary Fig. 4f). Similar random insertions of retrotransposons have been reported in humans^36^. Taken together, our data indicate that SV hotspots at piRNA loci in chickens, mice, and humans are formed independently, resulting from distinct mutational mechanisms (Fig. 4c). Thus, convergent evolution leads to the co-occurrence of SV hotspots and pachytene piRNA loci in the genomes of both birds and mammals.

### Silencing active TEs is a conserved function of pachytene piRNAs

**Fig. 5:**
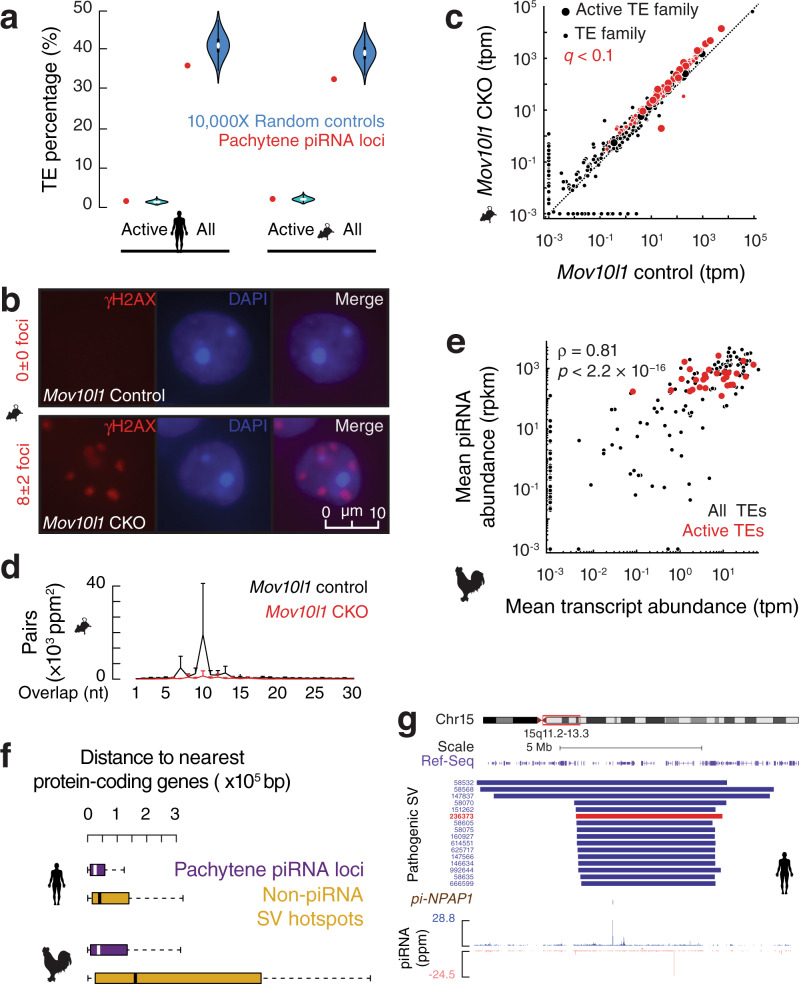
Silencing active TEs is a conserved function driving pachytene piRNA evolution. **a** The percentage of active TE sequences and total TE sequences in piRNA loci (red) and in randomly shuffled control sequences (aquamarine). Human *n* = 88, and mouse *n* = 100. Violin plots represent 10,000 randomly shuffled control sequences. **b** Immunofluorescence labeling of mouse round spermatids. γH2AX, marker for double strand breaks. The foci numbers were quantified from 90 round spermatids from three biological replicates. Scale bar, 10 µm. **c** Scatter plot of mean TE transcript abundance in *Mov10l1* CKO mutants versus that of littermate controls (*n* = 3). Each filled circle represents a TE family. Red, *q* value < 0.1. Each large circle represents an active TE family. Tpm transcript per million. **d** The 5′-5′ overlap between sense and anti-sense piRNAs mapping to TEs that are significantly increased in *Mov10l1* CKO mutants. Data are mean ± standard deviation (*n* = 3). Ppm parts per million. **e** Scatter plot of mean TE transcript abundance in 19 chickens from the 6 breeds versus mean TE piRNA abundance in 23 chickens from the 6 breeds. 30 active TE families (red). Rpkm reads per kilobase pair per million reads mapped to the genome. *p* value was calculated by Spearman’s rank correlation coefficient statistical test. **f** Box plots of the distance between SV hotspots and nearest protein coding genes in (upper) humans (piRNA *n* = 88, SV minus piRNA *n* = 269) and in (lower) chicken macrochromosomes (piRNA *n* = 779, SV minus piRNA *n* = 26). We only calculated the distance on macrochromosomes including chromosome Z in chickens where most of the piRNA loci localized (751/1321) because the assembly of microchromosomes has not been completed. *p* value is smaller than the threshold we can compute. Box plots show the 25th and 75th percentiles, whiskers represent the 5th and 95th percentiles, and midlines show median values. **g** Example of a pachytene piRNA locus overlapping with 16 SVs deposited in ClinVar. From top to bottom: RefSeq, pathogenic SVs (each SV is labeled by its Variation ID, and Red is associated with autism spectrum disorder), and piRNA reads from adult human testes (Blue represents Watson strand mapping reads; Red represents Crick strand mapping reads).

To identify the common selective pressure that drives convergence, we revisited the broadly accepted notion that mammalian pachytene piRNAs function beyond TE silencing. This notion is derived from the significantly lower fraction of TE sequences found at piRNA loci (~20%) compared to the bulk genome with 30–50% TEs^7,37^. We reason that the lower fraction may be due to the high recombination rates of the SV hotspots rather than a lack of function in TE silencing. Indeed, active TEs in mice and humans (young and actively transposing, all belong to retrotransposons) are not depleted from pachytene piRNA loci compared to the rest of the genome with a fraction of 1.6% in mice and 1.0% in humans (Fig. 5a, human or mouse piRNA loci vs. a set of control sequences by randomly shuffling piRNA loci on the same chromosome, one-tailed permutation test, *p* ≥ 0.07) despite a general depletion of all TE fractions (*p ≤* 2.6 × 10^−2^) from piRNA loci. We, therefore, tested whether the small fraction of TE-piRNAs encoded by mammalian pachytene piRNA loci are required for TE silencing. To avoid affecting pre-pachytene piRNAs and avoid potential piRNA-independent effects of PIWI gene knockout^38^, we conditionally knocked out (CKO) the mouse piRNA biogenic gene, *Mov10l1*, in spermatocytes driven by *Neurog3-cre*^39,40^, which specifically abolishes pachytene piRNAs without affecting the piRNAs expressed at earlier stages^41^. We confirmed the previous findings that this mutant proceeds through meiosis normally (Supplementary Fig. 5a) and arrests at the round spermatid stage with 8±2 γH2AX foci in the *Mov10l1* mutant, whereas wildtype round spermatids lack any foci (Fig. 5b, one-tailed Student’s t-test, *p* < 2.2 × 10^−16^). The increased γH2AX foci detected at the round spermatid stage were attributed to TE-independent DNA damage, as no significant increase in TE expression was detected by qPCR^41^.

Using RNA-seq, we found that while the expression of 83% of all TE families (1020 out of 1223) did not change, the expression of most active TE families (21 out of 24; 88%) was significantly increased in the *Mov10l1* CKO testes (Fig. 5c, large dots, *q* < 0.1)^42–46^. The derepression of active TEs was likely not detected in previous qPCR analyses^41^ because they were low throughput, using qPCR primers from TE consensus sequences that cannot distinguish active TEs from inactive TEs. Analysis of piRNAs from *Mov10l1* CKO testes revealed a significant decrease in piRNA-guided cleavage targeting these TEs, as measured by Ping-Pong signatures (Fig. 5d, one-tailed Student’s t-test, *p* = 3.5 × 10^−2^), indicating that TE derepression in *Mov10l1* CKO testes is due to a loss of piRNAs that target TEs. These TE-piRNAs are pachytene piRNAs because they are turned on during the pachytene stage and depleted in *Mov10l1* CKO mutants (Supplementary Fig. 5b), indicating that an essential role for the rare fraction of pachytene piRNAs to silence active TEs commences at the pachytene stage (Supplementary Fig. 5c).

To test whether silencing active TEs is also a function of chicken pachytene piRNAs, we developed a bioinformatics pipeline to define novel TE transpositions. Among the 13,442 insertion events detected using ONT-sequencing (Supplementary Fig. 6) we identified 30 active TE families in chickens. All belong to retrotransposons, comprised of 29 endogenous retroviruses (ERVs) and one Chicken Repeat 1 (CR1, LINE superfamily). These TE families are abundantly transcribed, as detected by RNA-seq (Fig. 5e), and are translated in testes, as demonstrated by Ribo-seq (Supplementary Fig. 7a). Consistent with their genomic polymorphisms (Supplementary Fig. 7b), the 30 active TE families displayed significantly higher expression variation among individuals than inactive TE families that are not transposing but still expressed in testes (Supplementary Fig. 7c). These 30 active TE families have invaded the chicken genome recently (Supplementary Fig. 7d, e), with an estimated median of 21.5 million years ago. Although only 2.4% of chicken pachytene piRNA loci encode active TEs, all active TE families are robustly targeted by pachytene piRNAs (Fig. 5e and Supplementary Fig. 7f, g). Therefore, silencing active TEs, commencing at the pachytene stage of germ cell development, is a conserved function for pachytene piRNAs. As active TEs are widespread^47^, young in the genome^26,48,49^, and highly detrimental without control^49^, the lineage-specific positive selection to silence active TEs acts as the second conserved force, together with the high level of local SV mutations that provide the evolutionary “substrate”, driving rapid divergence of pachytene piRNAs across amniotes to counter retrotransposon invasion and variation (Supplementary Fig. 5c).

### An adaptive balance between providing piRNA variations and detrimental SVs

In humans, SVs have been implicated in a number of heritable diseases, such as developmental delay, schizophrenia, and autism^50–54^. In both humans^27^ and chickens (Supplementary Fig. 8a), SVs are mostly depleted in protein-coding regions, indicating their deleterious impact on protein function. To understand why SV hotspots localizing at piRNA loci have not been eliminated by negative selection, we hypothesized that either the location of piRNA loci shields protein-coding genes from SVs or the function of generating polymorphic piRNAs balances the detrimental effects of SVs. To distinguish between these two possibilities, we asked whether piRNA loci are safe havens far away from protein-coding genes. While human SV hotspots are reported to localize at gene-poor regions^33^, we found that in both humans and chickens, protein-coding genes localize as close to piRNA loci as they do to randomly shuffled control sequences (Supplementary Fig. 8b). Compared to other SV hotspots that do not produce piRNAs, we found that piRNA loci are significantly closer to protein-coding genes in both humans and chickens (Fig. 5f, piRNA loci vs. a set of control sequences by randomly shuffling piRNA loci on the same chromosome, one-tailed permutation test, *p* = 1.0 × 10^−3^ and *p* = 2.9 × 10^−3^), indicating that SVs originated from piRNA loci are more likely to impair protein functions than other SV hotspots. To test whether the SVs originating from piRNA loci have any biomedical consequence, we annotated the 1349 *de novo* pathogenic SVs deposited in the ClinVar database from patients with substantial developmental and cognitive disorders (such as autism spectrum disorder). We found that these *de novo* pathogenic SVs, which originate in germ cells as they are usually too devastating to pass down to offspring, overlap with human pachytene piRNA loci significantly more than expected by chance (Fig. 3e, left, piRNA loci vs. a set of control sequences by randomly shuffling piRNA loci on the same chromosome, one-tailed permutation test, *p* = 8.5 × 10^−3^). The multiple pathogenic *de novo* SVs on 15q provide a compelling example for this overlap (Fig. 5g). Consistent with their pathogenic effects, we found that these SVs are only enriched in young piRNA loci (Supplementary Fig. 4a, right, piRNA loci vs. a set of control sequences by randomly shuffling piRNA loci on the same chromosome, one-tailed permutation test, *p* < 1.0 × 10^−4^), suggesting that they will not survive long-term selection. Our results suggest that pachytene piRNA loci are more deleterious than other SV hotspots and that the function of SV hotspots in protecting genome integrity by generating novel piRNAs makes the pathogenetic effects of SVs originating from piRNA loci in the soma tolerable.

To test whether the selection for silencing active TEs maintains deleterious pachytene piRNA loci over evolutionary time, we analyzed the 17,789 historical SVs identified by comparing great ape genomes with human genomes^55^. Although these genomic regions no longer generate SVs in the human population, these regions have generated SVs in the common ancestors of humans and great apes. We found that these historical SV regions are not enriched for pachytene piRNA loci (Fig. 3e, right, piRNA loci vs. a set of control sequences by randomly shuffling piRNA loci on the same chromosome, one-tailed permutation test, *p* = 0.14). We compared the active TE fractions in historical SV regions with those in current human SV hotspots and found that historical SVs (deletions and inversions in great apes) are completely depleted of active TEs with a median number of 0%, while current human SV hotspots harbor a median number of 1.5% active TEs (Supplementary Fig. 8c, historical SV regions vs. current SV hotspots, two-tailed Wilcoxon signed rank test, *p* < 2.2 × 10^−16^). Thus, our result suggests historical SV hotspots that are no longer able to produce piRNAs to silence active TEs have been eliminated during human evolution. Furthermore, considering that 30 active TE families invaded the chicken genome after chickens and ducks diverged, the negative selection explains why ancient piRNA loci in their common ancestors no longer exist, otherwise piRNA loci targeting ancient TEs would accumulate. Thus, negative selection to purge deleterious SV hotspots acts as the third conserved force that underlies the rapid turnover of piRNA loci (Supplementary Fig. 5c).

## Discussion

Here, using comparative genomic approaches, we have uncovered three forces underlying the rapid evolution of mammalian piRNA loci: high mutation rate, positive selection, and negative selection, reminiscent of the forces driving T cell and B cell maturation, whose maturation undergo VDJ recombination, positive selection for binding to their ligands, and negative selection for binding to self-antigens. While it is believed that TE surveillance mechanisms are set up prior to the pachytene stage through pre-natal and pre-pachytene piRNAs, DNA methylation, and histone modification in mammals^37,56–58^, we show that, upon the threat of active TEs, these early protection systems require reinforcement after the pachytene stage. Recent mapping of meiotic DSB sites in mice revealed the presence of active TE families at meiotic DSB hotspots^59,60^. The extensive DSB repair-mediated DNA synthesis during meiosis, together with the global replacement of histones with germ-line specific histone variants, transition proteins, and protamines during spermiogenesis, will modify the epigenetic factors present on TEs, potentially interrupting the early protection systems to call for a continuous requirement for silencing active TEs throughout spermatogenesis, ensuring the protection of the germline genome. The relative depletion of TEs at mammalian pachytene piRNA loci^7^, which would seem to argue against this essential function, may arise for three reasons. First, given that recombination drives genome contraction^61^, pachytene piRNA loci have a higher chance of eliminating TE sequences through recombination compared to the rest of the genome. Second, given that pachytene piRNA loci in mammals are enriched with SDs, their further enrichment with TEs make them excessively unstable, resulting in their elimination through purifying selection. Third, considering the old TEs can be repurposed to function in hosts^62^, the depletion allows transcripts containing TE fragments to be stably expressed commencing at the pachytene stage. Thus, the small fraction of piRNAs that target active TEs drive the evolution of pachytene piRNA loci in the same way that the small fraction of exonic regions drive the evolution of protein-coding genes.

We discovered a higher local mutation rate as a common mechanism underlying rapid adaptation of piRNA loci across amniotes (Supplementary Fig. 5c). While piRNA sequences are known to undergo positive selection to counter TE sequences^63–66^, positive selection, which fixes rare and beneficial mutations faster than neutral mutations, is not the only reason for the rapid evolution of mammalian piRNAs. Prior to our study, two mutational mechanisms of piRNA loci have been reported: (1) novel TE insertion into existing piRNA loci; and (2) copy number variations of existing piRNA loci^5,9^. The production of piRNAs from SV hotspots integrates these two mutational mechanisms and explains rapid piRNA birth, divergence, and loss. The high local mutation rate at piRNA loci benefits the function of piRNAs in silencing TEs, as illustrated in the following five scenarios. First, a more diverse piRNA pool can target TEs with mutation variants. Second, deletions that create TE truncations will disable the ability of TEs to recombine out of piRNA loci, thus trapping the truncated TE sequences in the loci to serve as “non-self memories”. For example, piRNAs targeting avian leukosis virus are produced from a truncated provirus in the White Leghorn genome^26^. Third, inversions generate anti-sense TE-piRNAs from the previous sense transcription orientation. The switch from sense to anti-sense TE-piRNAs has been proposed to be an important step for koalas to silence the KoRV-A gammaretrovirus^48^. Fourth, SVs can break genetic linkages between piRNAs with detrimental off-target effects and essential piRNAs on the same precursors, thus allowing segregation and selection against the detrimental piRNA. Fifth, a deleterious piRNA locus will be lost during evolution when the TEs targeted by piRNAs are no longer active. Given the continuous invasion of new TEs, without these elimination mechanisms, pachytene piRNA loci will not only unnecessarily accumulate given that other silencing mechanisms will eventually catch up to silence ancient TEs but also would increase detrimental off-target effects.

Our study provides an example of convergent evolution generating a novel organization between two unrelated and seemingly conflicting processes, genetic instability and defense systems protecting genome integrity. While piRNAs provide the main defense against TEs, we do not know how they keep up with the TEs. Compared to a conserved mechanism that works directly, natural selection acts gradually over evolutionary time scales. Under arms race pressure to defend against active TEs, the prevalence of piRNA loci localizing on SV hotspots is selected for, and, through convergent evolution, a common strategy is generated by conscripting “trouble makers” into “weapon creators” across amniotes. Our study argues against a conserved mechanism where all piRNA sequences are descendants/paralogs of piRNA loci from a common ancestor and instead indicates that piRNA loci with TE-defense functionality have evolved independently.

We are also one step closer to understanding the function of the non-TE fraction of pachytene piRNAs. Because our studies have effectively rejected the possibility of pachytene piRNA loci undergoing neutral evolution, this raises three possible explanations for the ubiquitous and abundant presence of non-TE piRNAs among amniotes. First, the production of non-TE piRNA is necessary for the biogenesis or function of piRNA targeting active TEs. Second, the non-TE pachytene piRNAs have their own sequence-specific function. However, considering the stringent target recognition rules of piRNAs^67^, the second possibility is unlikely to be true unless some auxiliary factors or unique subcellular environments loosen the pairing rules, which would further raise the challenge to distinguish self vs. non-self RNAs. Third, it is too expensive or difficult to eliminate these non-functional piRNAs. However, considering that the force to contract the genome is much stronger in avians, the extensive presence of non-TE piRNAs across amniotes is unlikely to be neutral. Further population genetics together with molecular biology is required to better distinguish these possibilities.

Finally, using comparative studies involving non-mammalian vertebrates whose genome evolution is distinct from humans, we show that the function of SV hotspots to protect germ-line genome integrity counterbalances the detrimental effects of SVs in somatic cells. This is predicted by the germ-soma conflict theory, which proposes that any advantages aiding the survival of germ cells would outweigh the deleterious effects in somatic cells^68^. Our work uncovers the principles driving the SV distribution. While previous studies on SVs focused mainly on identifying their impact on protein-coding genes^35,69–73^, our finding, which identified the adaptive function of nearly a hundred SV hotspots, argues for a paradigm shift from a gene-centric to a TE-centric view of genome evolution.

## Methods

### Animals

All experiments were reviewed and approved by the University of Rochester’s University Committee on Animal Resources, performed in a PHS Assured and AAALAC, Int. accredited facility, and the study is compliant with all relevant ethical regulations regarding animal research. The animal use protocol for sampling two indigenous village breeds, Tibetan chickens and Lvyang Blackbone chickens, were approved by the Animal Use and Care Committee of Northwest A&F University. The Athens Canadian Random Bred (ACRB) animals were raised under standard broiler and broiler breeder conditions under the protocol (18083) approved by the International Animal Care and Use Committee (IACUC) at the University of Arkansas. White Leghorn Cornell Special C strain was raised and euthanized under the IACUC at Cornell University. Araucana was purchased from SkyBlueEgg Araucana (Winnfield, LA) and Awesome Araucana chicken hatchery (Redding, CA). Pekin duck testes were purchased from a local farm (LeRoy, NY). Rooster testes from Red Jungle Fowl were collected from Hopkin Avian facility at UCD under the protocol #20591.

### Histology and immunostaining

For histologic analysis, testes were fixed in Bouin’s solution overnight, embedded in paraffin, and sectioned at 4 µm. Following standard protocols, sections were deparaffinized, rehydrated, and then stained with Hematoxylin and Eosin.

Immunostaining was performed on squashed spermatocytes and spermatids as previously described^74^. Seminiferous tubules were fixed in 2% paraformaldehyde containing 0.1% Triton X-100 for 10 min at room temperature, placed on a slide coated with 1 mg/ml poly-L-lysine (Sigma) with a small drop of fixative, gently minced with tweezers, and squashed. The coverslip was removed after freezing in liquid nitrogen. The slides were later rinsed three times for 5 min in PBS and incubated for 12 h at 4 °C with rabbit anti-CIWI antibody (1:100 dilution; Proteintech, 15659-1-AP), rabbit anti-SYCP1 antibody (1:100 dilution, Thermo Fisher, PA1-167630), or rabbit anti-γH_2_AX (1:250 dilution; Millipore, 05-636-1). Secondary antibodies conjugated with Alexa Fluor 488 (Molecular Probes, Eugene, OR, USA) were used at a dilution of 1:500.

### RNA-sequencing

Strand-specific RNA-seq libraries were constructed following the TruSeq RNA sample preparation protocol as previously described^17^. Ribosomal RNAs (rRNAs) were depleted from total RNAs with complementary DNA oligomers (IDT) designed for chicken rRNAs and RNase H^75^.

### Small RNA sequencing library construction

Small RNA libraries were constructed and sequenced as previously described^40^, using oxidation to enrich for piRNAs by virtue of their 2´-*O*-methyl-modified 3´ termini.

### Ribo-seq library construction

Ribosome profiling was performed as previously described^26^. After RNase treatment, testis lysates were loaded on a 10–50% (w/v) linear sucrose gradient and after centrifugation the fractions corresponding to 80S monosomes were recovered. rRNA fragments were removed as previously described^75^.

### General bioinformatics analyses

Analyses were performed using piPipes v1.4^76^. All data from the small RNA-seq, RNA-seq, Ribo-seq, and genome sequencing were analyzed using the latest chicken genome release GCA_000002315.5, Pekin duck genome release (ZJU1.0 GCA_015476345.1), mouse genome release mm10 (GCF_000001635.7), and human genome release hg38 (GCF_000001405.27). Generally, one mismatch was allowed for genome mapping and three mismatches were allowed for transcriptome mapping. Chicken TE families were updated according to Repbase^77^, with a total number of 245 consensus sequences. For small RNA analysis, the transcriptome included the 245 TE families and 1321 piRNA clusters. For RNA-seq, the transcriptome included mRNAs, lncRNAs, piRNA loci, tRNAs, and TE families. Supplementary Data 1 reports the statistics for the high-throughput sequencing libraries constructed in this study.

For small RNA sequencing, libraries were normalized to the sum of total miRNA reads with the assumption that total miRNA abundance remains constant during spermatogenesis, according to the expression level of Argonaute genes (Supplementary Fig. 1b). Oxidized samples were calibrated to the corresponding total small RNA library using the abundance of shared piRNA species. Genome mapping reads >23 nt were selected for further piRNA analysis. The piRNA abundance per TE or per piRNA locus is reported either as parts per million reads mapped to the genome (ppm) or reads per kilobase pair per million reads mapped to the genome (rpkm) using a pseudo count of 0.001. We analyzed previously published small RNA libraries from wild-type mouse testes at 10.5 dpp (GSM1096582), 12.5 dpp (GSM1096584), 14.5 dpp (GSM1096584), 17.5 dpp (GSM1096585), and 20.5 dpp (GSM1096586); from the testes of *Mov10l1* CKO mouse mutants (GSM4160774, GSM4160775, GSM4160776, GSM4160777, GSM4160778 and GSM4160779) and littermate controls at adult stage (GSM4160768, GSM4160769, GSM4160770, GSM4160771, GSM4160772, and GSM4160773);^40^ and from human testes (GSM4030214 to GSM4030227) at adult stage^7^.

For RNA-seq reads, the tpm (transcripts per million) value was quantified using the Salmon algorithm^78^. The tpm value with a pseudo count of 0.01 was used for all analyses. We analyzed the published RNA-seq libraries from *Mov10l1* CKO mutants (GSM4160761, GSM4160762 and GSM4160753) and littermate controls (GSM4160758, GSM4160759 and GSM4160760)^40^.

Ribo-seq analysis followed the modified small RNA pipeline, including the junction mapping reads as previously described^40^. Uniquely mapping reads between 26 nt and 32 nt were selected for further analysis.

Statistical analyses were performed in R 3.5.0^79^. The significance of the differences was calculated by Wilcoxon rank-sum test unless otherwise indicated. Box plots show the 25th and 75th percentiles, whiskers represent the 5th and 95th percentiles, and midlines show median values.

### Clustering and heatmap

SV data were converted to a “Yes” or “No” table. Then, we applied the hkmean function from the factoextra package in R to conduct Hierarchical K-Means clustering of our data. The clustering number was passed to the pheatmap function with parameter annotation_row to generate the final clustering figure. At the same time, the pheatmap function was directly used to perform hierarchical clustering of our data (parameter cluster_rows=T, clustering_method = “complete”).

### Identifying SV hotspots

To identify the SV hotspots, we followed the previous methods used in human SV hotspots identification^27^ with the following modifications. We combined all the SVs (tandem duplication, inversion, deletion and insertion) and used the “hotspotter” function from the primatR package^80^ (num.trial=1000, *p*-value ≤ 0.001). We optimized the bw parameters with 200, 2000, 20,000, 200,000 and 2,000,000. The typical avian karyotype consists of a small number of relatively large macrochromosomes and many very small microchromosomes (chromosome <20 Mbps)^23,81^. In chickens (Fig. 1c), in addition to sex chromosomes, autosomes can be further classified based on size into macrochromosomes (Chr1–5, 58% of the rooster genome), intermediate chromosomes (Chr6–12, 18% of the genome), and microchromosomes (Chr13–38, 16% of the genome). We found that the bw=2000 parameter provides the most parsimonious output to explain the most SVs with the least genomic regions for macrochromosomes and intermediate chromosomes. The optimal parameter is bw=200 for microchromosomes and unassigned contigs and is bw=20,000 for sex chromosomes. We ended up with 192 SV hotspots.

### Nucleotide periodicity

Nucleotide periodicity was computed as previously described^40^. We first aligned the ribosome protected fragments (RPFs) to each other using 5´-end overlap analysis and reported the distance spectrum. An annotated ORF was not a prerequisite for this analysis as the distance spectrum of RPFs from mRNAs already showed a 3-nt periodicity pattern. We then transformed the distance spectrum using the “periodogram” function from the *GeneCycle* package^82^ with the “clone” method. The relative spectral density was calculated by normalizing to the value at the first position.

### Defining novel TE transposition

We built a bioinformatics pipeline to define novel TE transpositions using the 13,442 insertion events identified from ONT-sequencing in all six chicken breeds (Supplementary Fig. 6a). We assembled the insertion sequences using supporting reads at each insertion and aligned them to 245 consensus sequences of chicken TE families deposited in Repbase^77^ using BLAST^83^ with an e-value cutoff of 10^−10^. We further filtered sequences based on >80% sequence identity, >80% alignment length, and <20% gaps, the 80-80-80 rule used in TE identification^84^. To define transposition-induced insertions, we required insertions to contain complete 5´ and 3´ ends of DNA transposons and LTR retrotransposons (ERVs). As for insertions derived from non-LTR retrotransposons, only complete 3´ends were required due to the prevalence of 5´ truncation during target-primed reverse transcription^85^. In total, we identified 644 putative TE-induced integration sites.

Although Red Jungle Fowl are commonly referred to as the “ancestor” of domestic chickens^86^, they have continued to evolve for thousands of years post-domestication. Therefore, the detected insertion events could theoretically be either novel insertions (Supplementary Fig. 6b, red shading) or ancestral deletions that do not reflect active TEs (Supplementary Fig. 6b, gray shading). Nonetheless, we can distinguish between the two possibilities given that transposition events involve the precise insertion of TE consensus sequences, whereas deletion events inevitably alter additional sequences. For example, the deletion of ERVs caused by recombination will leave solo-LTRs. Among 644 putative TE-induced insertion sites, 481 contain only solo-LTRs, whereas 185 contain intact ERVs and only 12 contain CR1 elements. The high number of insertion sites with solo-LTRs indicates a high recombination rate, which is consistent with the notion that despite the paucity of interchromosomal changes, intrachromosomal changes are common in birds^87^. For putative ERV transpositions, we applied two additional criteria to remove ancestral deletion events: (1) the insertions must start and end with TEs; and (2) intact ERV insertion sites should not map to solo LTRs in the reference genome (Supplementary Fig. 6c, top). Using these criteria, we identified 519 insertions from ERV families, including the insertion specifically in Araucana near *SLCO1B3* (solute carrier organic anion transporter family member 1B3), which has been shown to cause their blue eggshells (Supplementary Fig. 6c, bottom)^29^.

CR1 elements are long interspersed nuclear elements in chickens^88^ that represent the major non-LTR retrotransposons in birds^89^. Arising before the divergence of birds and reptiles^90^, CR1 elements are believed to exhibit little activity in most avian species including chickens^89^, as avian genomes harbor a paucity of retroposed pseudogenes^23^. However, it remains unclear whether CR1 is completely extinct in the chicken genome. Since the reverse transcription of CR1 starts from their 3´ ends, we reasoned that their 3´ends should be preserved at one end of the insertions, but their 5´ ends are likely truncated or mutated due to the dissociation of the reverse transcriptase from mRNA during reverse transcription. Thus, among the 12 putative transposition events harboring CR1, we removed 8 putative events that harbored the 3´ ends of CR1 in the middle of the insertions. For these 8 putative events, we were able to rule out the possibility of 3´ transduction that results in the co-transposition of flanking sequences along with a retrotransposon^91^, as the additional sequences in the insertions did not map to known sequences in the genome. The remaining four CR1 insertions all came from CR1-F2, a sub-family of CR1, and we detected full-length copies (~4.6 kb) of the CR1-F2 family in chicken genomes (Supplementary Fig. 6d). Thus, our data indicate that LINE-retrotransposons are quiescent rather than extinct in the chicken genomes.

### TE age estimation

By computing the average percent divergence of each TE family from their consensus sequences, we found the insertions from the 30 active TE families have a significantly lower divergence percentage than the insertions from inactive TEs (Supplementary Fig. 7e). Using a neutral substitution rate of ~1.9 × 10^−3^ substitutions per site per million years^22^ and a Jukes-Cantor 1969 model correction^92^, we estimated that these 30 active TEs invaded the chicken genome a median of 21.5 million years ago.

### Whole genome alignment between chicken and duck genomes

We used Minimap2 with parameter ‘-cx asm20’ to align the chicken genome to the duck genome. We then performed the LiftOver function to detect the homologous regions of chicken pachytene piRNA loci on duck genomes using paftools^93^.

### Ping-Pong analysis

Ping-Pong amplification was analyzed by the 5′–5′ overlap between piRNA pairs from opposite genomic strands^26^. Overlap scores for each overlapping pair were the product of the number of reads of each of the piRNAs from opposite strands. The overall score for each overlap extension (1–30) was the sum of all such products for all chromosomes. Heterogeneity at the 3′ ends of small RNAs was neglected. The Z-score for a 10 bp overlap was calculated using the scores of overlaps from 1–9 and 11–30 as background.

### Rooster piRNA-producing loci detection

We used the same dynamic programming algorithm that we developed previously^17^ to identify genomic regions with the highest piRNA density. All oxidized small RNA reads (> 23 nt) from diverse breeds and different developmental stages were used to define the chicken loci. We assumed that piRNA clusters comprise at most 5% of the chicken genome. We first split the genome into 1 kbp non-overlapping windows and computed piRNA abundance for each window. The mean of the top 5% of windows was used as the penalty score for the dynamic programming algorithm. The algorithm computes the cumulative piRNA abundance score as a function of the window index along each chromosome. The score at a window is the sum of the score in the previous window plus the piRNA abundance in the current window minus the penalty score, with negative scores being reset to 0. The maximum score indicates the largest piRNA cluster. We extracted the largest piRNA cluster, recomputed the scores at the corresponding windows, and searched for the next cluster. This process was continued iteratively until the scores for all windows were zero. The boundaries of each cluster were further refined by including those base pairs for which piRNA abundance exceeded the mean piRNA abundance of the top 5% windows. We required a piRNA cluster to have at least 1 unique mapping read. The coordinates of all 1321 piRNA loci are reported in Supplementary Data 1.

### Genomic repeat annotation

The current annotation of TEs in the chicken genome deposited in the UCSC genome browser was performed on February 01, 2017 using the Repbase library released on January 27, 2017 and thus is outdated. We used the search for the occurrence of the latest 245 TE family consensus sequences from Repbase^77^ in the chicken genome using a homology-based method proposed by the RepeatMasker program^94^. The 523 TE integration sites involve 21 TE families, including 20 ERV families and CR1-F2. Among the 20 ERV families, 12 families are solo LTRs with no internal sequences detected in the insertions, likely due to efficient intrachromosomal recombination. Upon retrieving their Internal sequences from Repbase^77^, where they are annotated as separate TE families, we identified a total of 30 active TE families. Since not all the TEs are translated, we removed the DNA transposons, SINEs, and solo LTR transposons from the translation analysis.

### ONT library construction and sequencing

To avoid PCR amplification bias, we constructed the ONT-seq libraries without PCR amplification, which also allows us to preserve DNA methylation patterns for future epigenetic analysis. We sequenced DNA purified from testes because of the large amount of tissue available and genetic and epigenetic alterations in testes have the potential to impact following generations. Genomic DNA was size selected using the Pippin HT DNA Size Selection System (Sage Science, Beverly, MA, USA) to enrich for molecules >10 kb. The DNA ends were repaired and dA-tailed using the NEBNext FFPE DNA repair Mix and NEBNext Ultra II End repair/dA-tailing module (New England BioLabs, Ipswich, MA, USA) according to the manufacturer’s instructions, and samples were cleaned up using AMPure XP beads (Beckman Coulter, Indianapolis, IN, USA). Then, samples were subjected to adapter ligation using the NEBNext Quick Ligation Module (New England BioLabs, Ipswich, MA, USA), according to the manufacturer’s instructions, and cleaned up again using the AMPure XP beads. The prepared libraries were then subjected to ONT sequencing on a SpotON flow cell (Oxford Nanopore Technologies, Oxford, UK). Flow cells were primed using the Flow Cell Priming Kit (Oxford Nanopore Technologies), and the libraries were prepared and loaded according to the Ligation Sequencing Kit (Oxford Nanopore Technologies). Lastly, flow cells were loaded into the PromethION P48 (ONT-08-00443-02) and run according to the relevant parameters.

### Quality control of ONT sequencing data

Raw data collected in this experiment were obtained as fast5 files, and after conversion of electric signals into base calls via the guppy (Oxford Nanopore Technologies, UK), the reads with mean qScore greater or equal to 7 were kept to continue subsequent bioinformatic analysis.

### Alignment and SV calling

The filtered ONT data were aligned with the chicken genome (galGal6) using NGMLR v0.27^95^ with ‘-x ont’. The SVs were detected using Sniffles v1.0.8^95^ with --report_BND --ignore_sd -t 4 -q 0 -n -1 -l 50 -s 2 --genotype’ and further required read numbers ≥ 10. We also independently used SVIM v1.4.2^96^ to call SVs with default settings, and SVs with score ≥ 10 were used for further analysis. We required the SVs of duplication, deletion, and inversion to be called by both Sniffles and SVIM.

### Shuffling test

We performed a shuffling test to determine whether the median distance between X’s and Y’s are significantly different from what we would observe if X’s are randomly distributed on the chromosome. We repeated the shuffling 10,000 times, and each time we calculated the median distance between the shuffled X’s and Y’s. We denoted the observed median by ${M}^{{{{{{\rm{obs}}}}}}}$ and the shuffled median by ${M}_{1},\ldots,{M}_{{{{{\mathrm{10,000}}}}}}$. The *P*-value of the shuffling test can be calculated as *P* = min{1, max{*P*_*l*_, *P*_*u*_}}, where ${P}_{l}={\sum }_{i=1}^{{{{{\mathrm{10,000}}}}}}1({M}_{i}\le {M}^{{{{{{\rm{obs}}}}}}})/{{{{\mathrm{10,000}}}}}$ and ${P}_{u}={\sum }_{i=1}^{{{{{\mathrm{10,000}}}}}}1({M}_{i}\ge {M}^{{{{{{\rm{obs}}}}}}})/{{{{\mathrm{10,000}}}}}$. Similar analyses were applied to test the overlapping numbers and conservation scores. Randomization was performed using bedtools shuffle function with restriction on the same chromosome^97^. The 861 chicken SDs were downloaded from ref. ^98^ and converted to galgal6 using liftover^93^. The 278 human SV hotspots were downloaded from ref. ^27^. The 1349 *de novo* human pathogenic SVs were deposited in the ClinVar database from patients with substantial developmental and cognitive disorders (such as autism spectrum disorder). The 18,036 breakpoints between humans and great apes were downloaded from ref. ^55^, and the 5892 regions that are deleted in great apes and 29 regions that are inverted in great apes compared to humans were used for active TE fraction analysis. The 62,038 human meiotic DSB hotspots were downloaded from ref. ^99^. The 3802 human SDs were downloaded from^34^. The 13,906 mouse meiotic DSB hotspots were downloaded from ref. ^60^. The 659,775 mouse SDs were downloaded from UCSC^100,101^.

### Variance analysis

We calculated expression variance as described^102^. For each TE family, piRNA locus, or SV, we calculated its median expression level and the coefficient of variation (CV) from the normalized read counts across individuals. We used the residuals from a locally weighted regression (LOESS) of the CV on median expression to obtain a measure of expression variation relative to the expected variation at a given expression level. The advantage of this method is that the expression variance is no longer correlated with the levels of the expression^102^. This method was applied to calculate the variance for strand bias and diversity of piRNAs.

### Diversity analysis

Small RNA species were counted and summarized into a matrix based on mapping to each TE family or SV. The Shannon diversity index was calculated by the diversity function from the vegan package under R v3.5.0. The final diversity value is based on each TE family or SV.

### Sequence complexity analysis

The Wootton–Federhen complexity score, *cwf*, was calculated as previously described^103,104^. The calculation is shown as the following formula where *N* is the length of the sequence and *n*_*i*_ is the total count of base *i*.

$${cwf}=\frac{1}{N}\times {\log }_{4}\frac{N!}{\mathop{\prod}\limits_{i\in {ACTG}}{n}_{i}!}$$

### Reporting summary

Further information on research design is available in the Nature Portfolio Reporting Summary linked to this article.

## Supplementary information

Supplementary Information

Peer Review File

Description of Additional Supplementary Files

Supplementary Data 1

Reporting Summary

## Data Availability

The data supporting the findings of this study are available from the corresponding author upon reasonable request. Next-generation sequencing data used in this study have been deposited at the NCBI Gene Expression Omnibus under the accession number GSE165330. Previously published datasets used in this study are available from GEO under following accession number: small RNA libraries from wild-type mouse testes at 10.5 dpp (GSM1096582), 12.5 dpp (GSM1096584), 14.5 dpp (GSM1096584), 17.5 dpp (GSM1096585), and 20.5 dpp (GSM1096586); from the testes of *Mov10l1* CKO mouse mutants (GSM4160774, GSM4160775, GSM4160776, GSM4160777, GSM4160778 and GSM4160779) and littermate controls at adult stage (GSM4160768, GSM4160769, GSM4160770, GSM4160771, GSM4160772 and GSM4160773); and from human testes (GSM4030214 to GSM4030227) at adult stage. The published RNA-seq libraries from *Mov10l1* CKO mutants (GSM4160761, GSM4160762 and GSM4160753) and littermate controls (GSM4160758, GSM4160759 and GSM4160760). The detailed statistics of high-throughput sequencing and related results have been summarized in Supplementary Data 1.
