# Supplementary material for: Amniotes co-opt intrinsic genetic instability to protect germ-line genome integrity
Source: Nat Commun. 2023 Feb 13;14:812. doi: 10.1038/s41467-023-36354-x (PMC9925758; doi:10.1038/s41467-023-36354-x)
Supplement: Supplementary file 1 — Supplementary Information [file 41467_2023_36354_MOESM1_ESM.pdf]

Supplementary Information

Amniotes co-opt intrinsic genetic instability to protect germ-line genome integrity

Yu H. Sun, Hongxiao Cui, Chi Song, Jiafei Teng Shen, Xiaoyu Zhuo, Ruoqiao Huiyi Wang, Xiaohui Yu, Rudo Ndamba, Qian Mu, Hanwen Gu, Duolin Wang, Gayathri Guru Murthy, Pidong Li, Fan Liang, Lei Liu, Qing Tao, Ying Wang, Sara Orlowski, Qi Xu, Huaijun Zhou, Jarra Jagne, Omer Gokcumen, Nick Anthony, Xin Zhao, and Xin Zhiguo Li

Correspondence: xin.zhao@mcgill.ca (X.Z.) and Xin_Li@urmc.rochester.edu (X.Z.L.)

This PDF file includes:

Supplementary Figs. 1-8

Supplementary Figures

Supplementary Fig. 1.

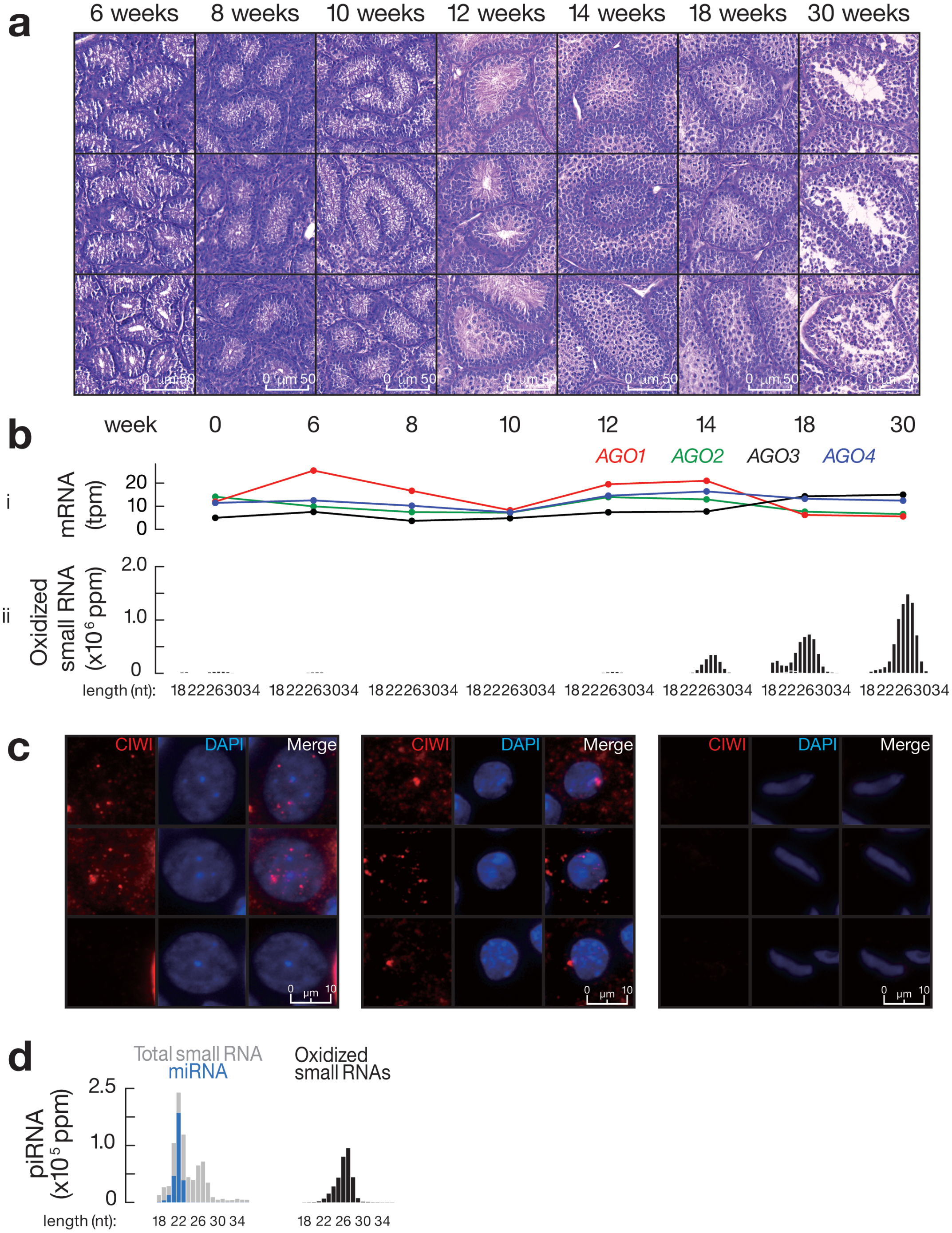

Supplementary Fig. 1. Pachytene piRNAs exist in avian species. (a) Histology of testis sections of ACRB harvested at different developmental time points shown by H&E staining. Scale bar, 50 µm. **(b)** A burst of piRNA expression during spermatogenesis. (upper) Expression of *AGO1, AGO2, AGO3, and AGO4* as measured by RNA-seq. The relatively stable expression of miRNA-binding proteins suggests constant miRNA levels during spermatogenesis. Tpm, transcript per million. (lower) Length distribution of oxidized small RNAs. Ppm, parts per million. **(c)** Immunolabeling of squashed testis cells from adult rooster testes that are not pachytene spermatocytes using anti-CIWI and DAPI. Scale bar, 10 µm. **(d)** Length distributions of testis small RNAs from adult ducks. Ppm, parts per million.

Supplementary Fig. 2.

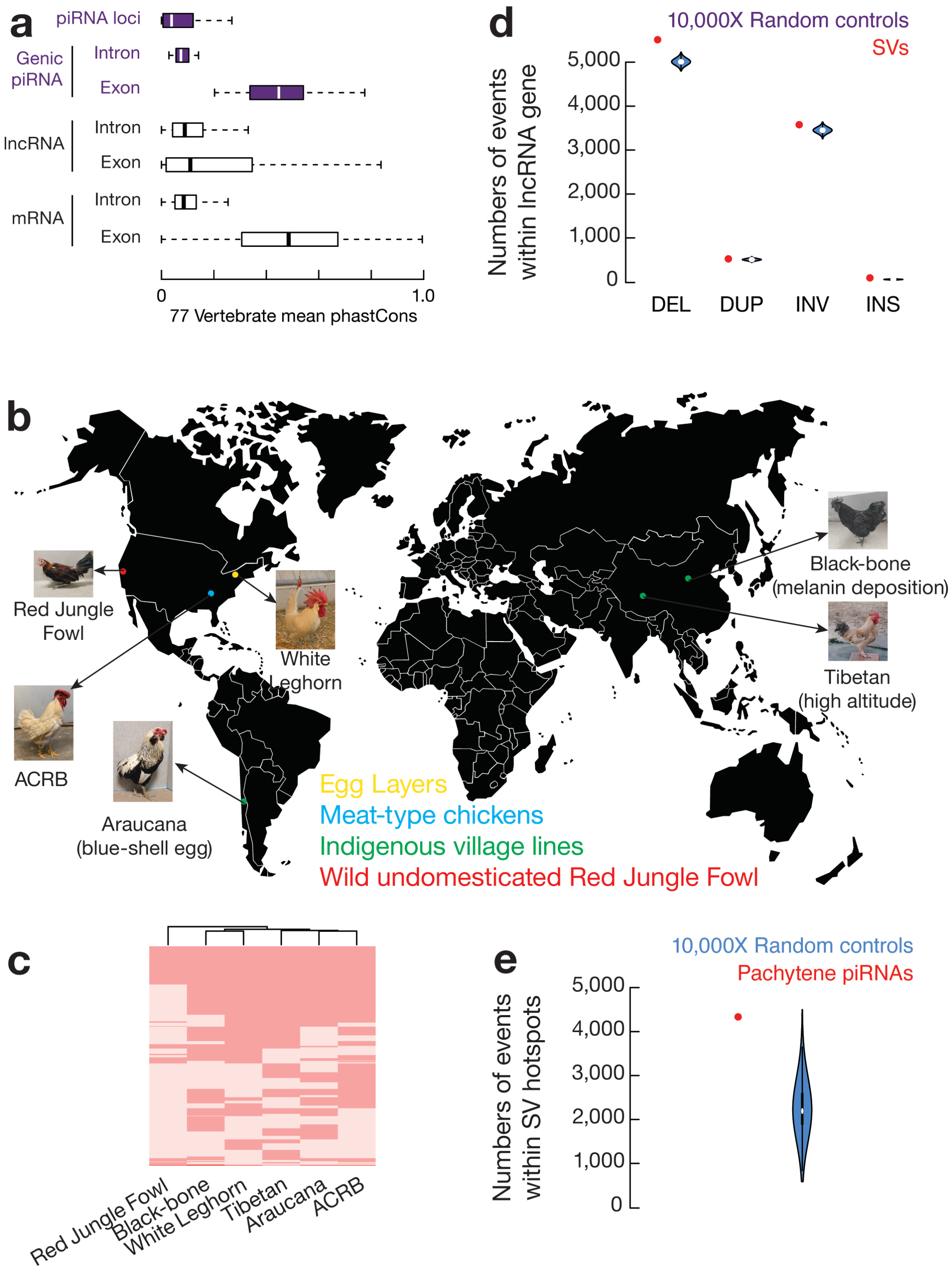

Supplementary Fig. 2. Chicken piRNA loci are SV hotspots. (a) Box plots of the mean phastCons score from the 77 vertebrate genome alignments (probability that each nucleotide belongs to a conserved element) of the genomic regions of introns and exons of lncRNAs (Intron n=20,789, Exon n=51,428), mRNAs (Intron n=27,823, Exon n=28,281), and piRNA producing mRNAs (n of intron and exon = 23), as well as intergenic piRNA loci. Box plots show the 25th and 75th percentiles, whiskers represent the 5th and 95th percentiles, and midlines show median values. **(b)** Geographic locations of the chicken breeds used in this study. **(c)** Heatmap of presence (dark pink) or absence (light pink) of each SV in each chicken. **(d)** The number of SVs (red, n of lncRNA gene = 51,428) and randomly shuffled control sequences (purple) falling into the piRNA loci. Violin plots represent the randomly shuffled control sequences that were computed 10,000 times. **(e)** Number of chickens pachytene piRNA loci (red) and randomly shuffled control sequences (aquamarine) overlapping with chicken SV hotspots (n = 192). Violin plots represent the medians of randomly shuffled control sequences that were computed 10,000 times.

Supplementary Fig. 3.

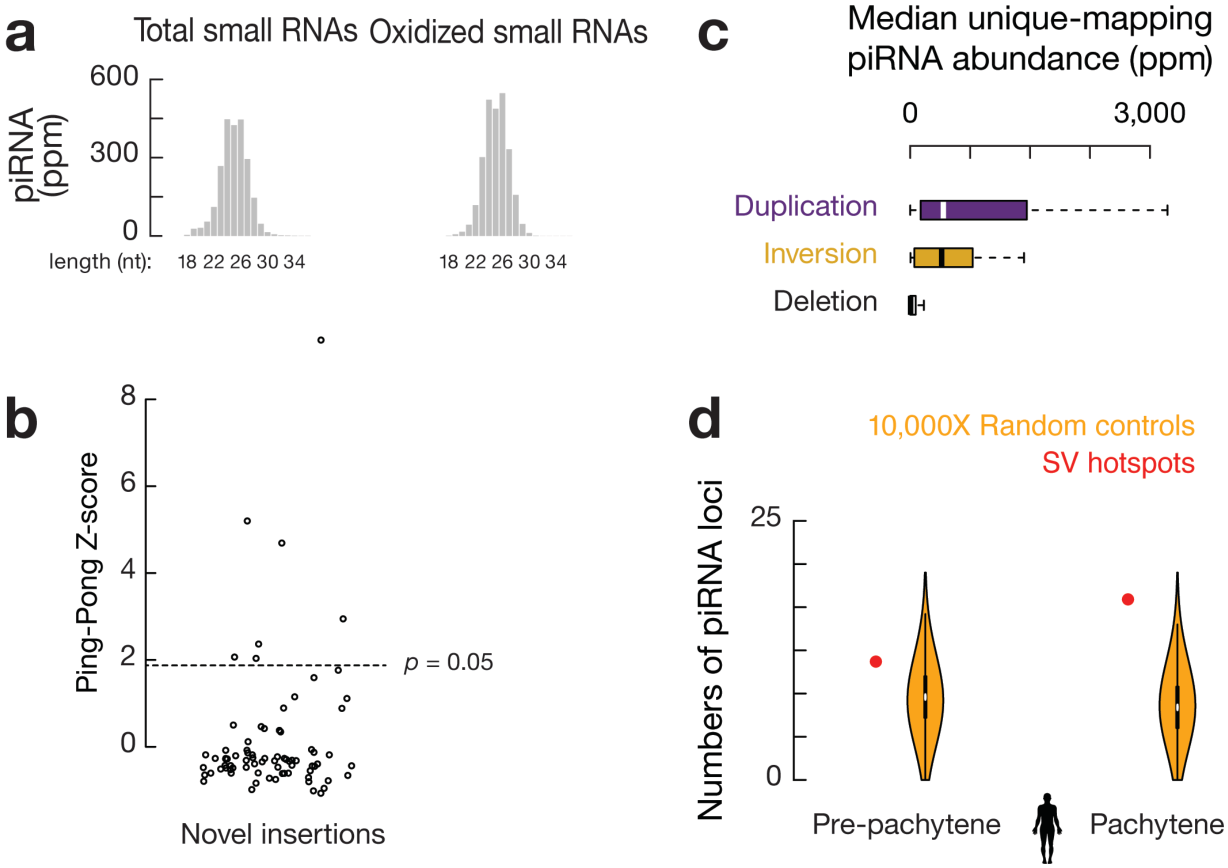

Supplementary Fig. 3. Polymorphic piRNAs within chicken species. (a) Length distributions of small RNAs mapping to novel insertions from adult testes of 23 chickens from the 6 breeds. **(b)** The Ping-Pong Z-score of piRNAs that comes from each insertion and does not map to the reference genome. The *Z*-score describes whether a significant ten-nucleotide overlap ("Ping-Pong") was detected. *Z*-score >1.96 corresponds to p-value < 0.05, p value was calculated with two-tailed *Z* test. **(c)** Box plots showing the median abundance of uniquely mapped piRNAs per SV among 23 chickens from 6 breeds. Box plots show the 25th and 75th percentiles, whiskers represent the 5th and 95th percentiles, and midlines show median values. **(d)** Number of human SV hotspots (red), their randomly shuffled control sequences (yellow) overlapping with human pre-pachytene piRNA loci (left, n=82), and pachytene piRNA loci (right, n=88). Violin plots represent the medians of randomly shuffled control sequences that were computed 10,000 times.

Supplementary Fig. 4.

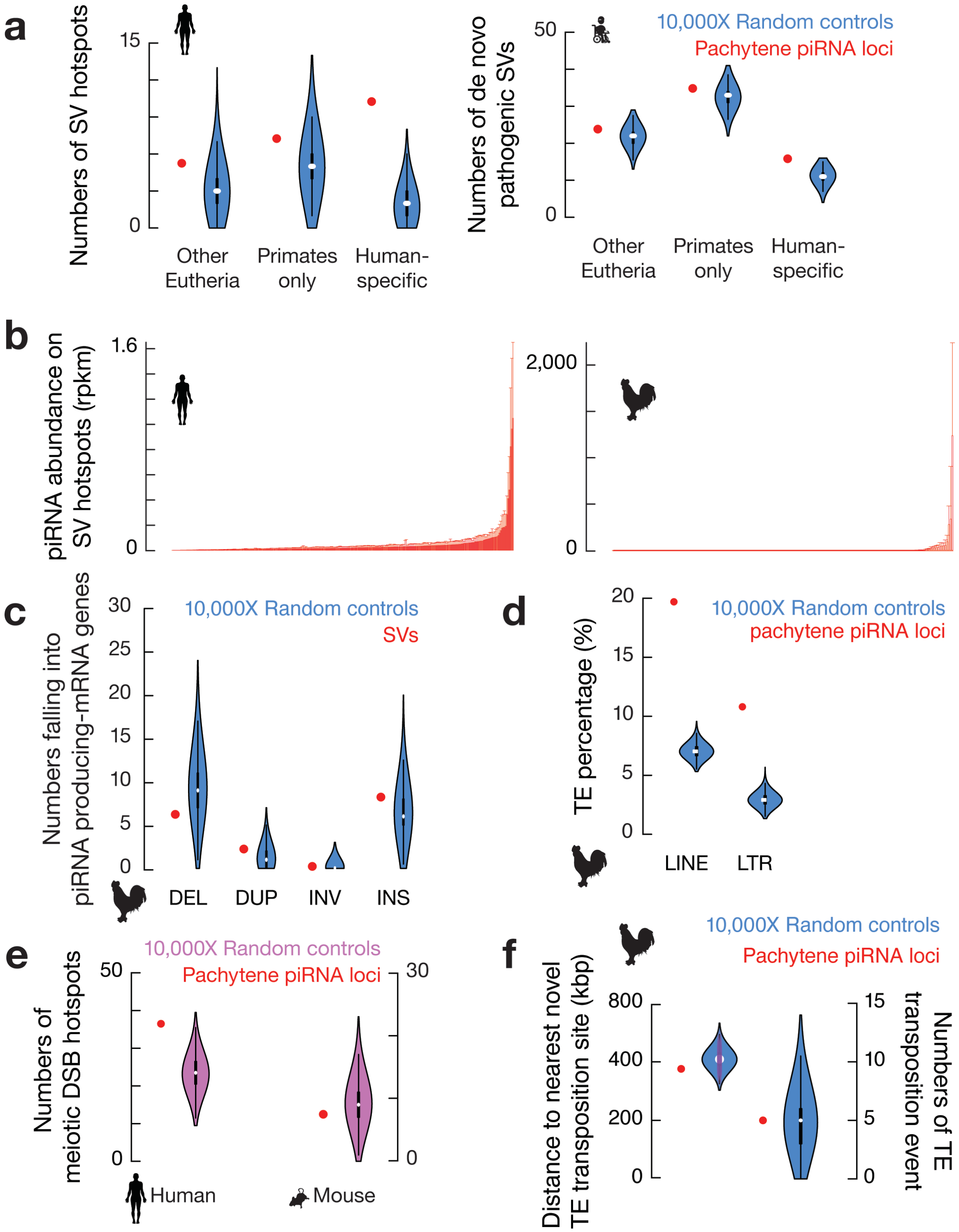

Supplementary Fig. 4. Diverse mutational mechanisms result in SV hotspots. (a) Number of human pachytene piRNA loci (red, Other Eutherian n=29, Primates only n=43, Human-specific n=16) and randomly shuffled control sequences (aquamarine) overlapping with SV hotspots within healthy human populations (left) and de novo pathogenic SVs detected in patients (right). Violin plots represent the medians of randomly shuffled control sequences that were computed 10,000 times. **(b)** Bar plots showing the piRNA abundance of SV hotspots in humans (left) and in chickens (right). Data are mean ± standard deviation (n=14 humans; n=23 chickens). Rpkm, reads per kilobase of transcript, per Million mapped reads. **(c)** The number of SVs (red) and randomly shuffled control sequences (blue) falling into the chicken piRNA producing mRNA genes (n= 23). Violin plots represent the randomly shuffled control sequences that were computed 10,000 times. **(d)** The percentage of TE sequences in piRNA loci (red, n = 1,321) and in randomly shuffled control sequences (blue). Violin plots represent the randomly shuffled control sequences that were computed 10,000 times. **(e)** Number of pachytene piRNA loci (red) and randomly shuffled control sequences (magenta) overlapping with meiotic DSB hotspots from humans or mice (human n=88, mice n=100). Violin plots represent the medians of randomly shuffled control sequences that were computed 10,000 times. **(f)** (Left) Median distance between novel TE transposition and nearest chicken piRNA loci (red, n=1,321) or nearest randomly shuffled control sequences (blue). (Right) Number of novel TE transposition events falling in chicken piRNA loci (red) and randomly shuffled control sequences (blue). Violin plots represent the numbers of randomly shuffled control sequences that were computed 10,000 times.

Supplementary Fig. 5.

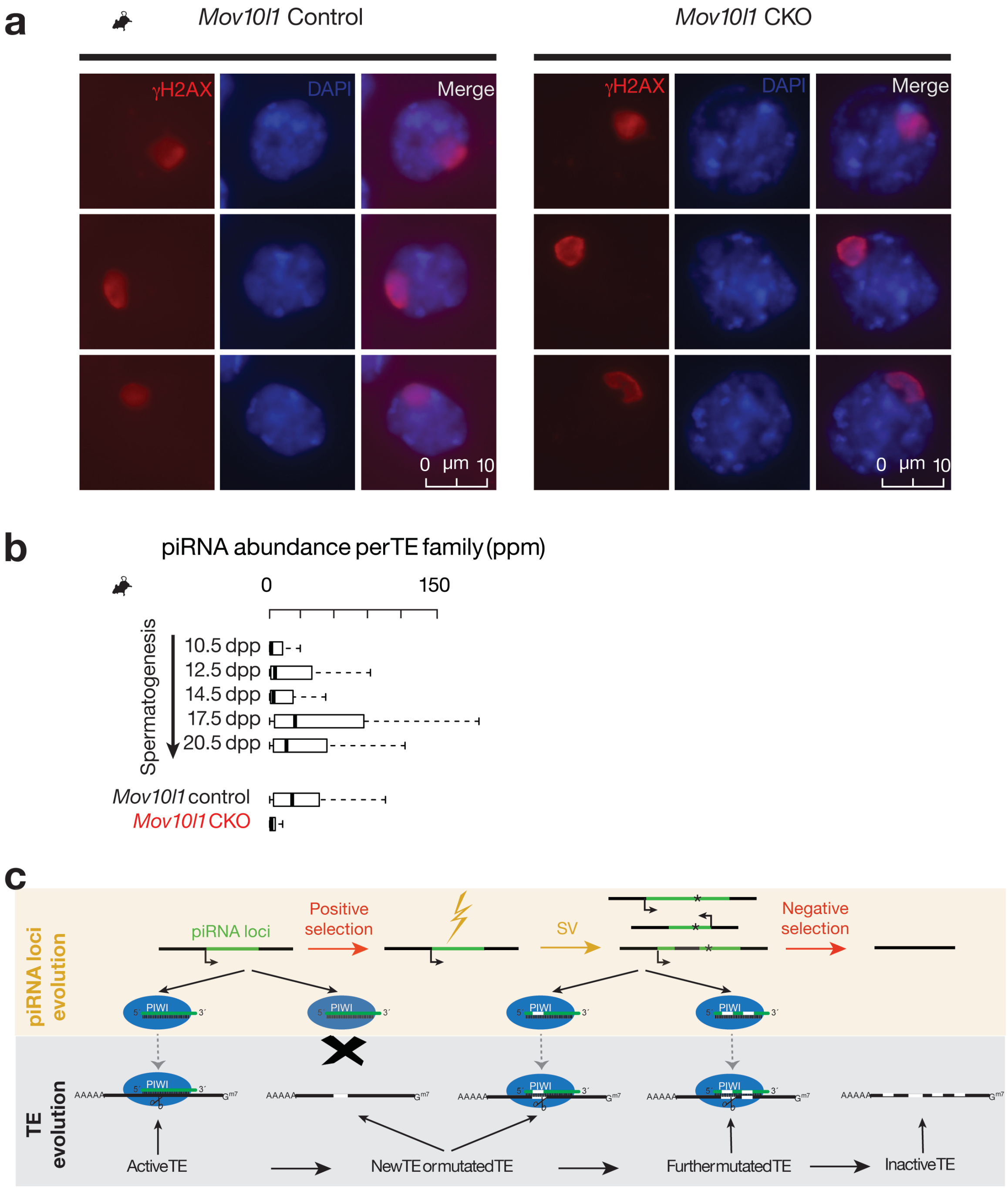

Supplementary Fig. 5. piRNA plasticity is selected for due to their essential function to suppress TEs. (a) Immunofluorescence labelling of mouse pachytene spermatocytes. gH2AX, marker for double strand breaks. Scale bar, 10 μm. **(b)** Box plots showing piRNA abundance per TE family (n =245) as spermatogenesis progresses. Ppm, parts per million. Box plots show the 25th and 75th percentiles, whiskers represent the 5th and 95th percentiles, and midlines show median values. **(c)** Model scheme showing high local mutation rate of SVs, together with positive selection and negative selection, drives the rapid adaptation to silence active TEs via generating novel piRNAs.

Supplementary Fig. 6.

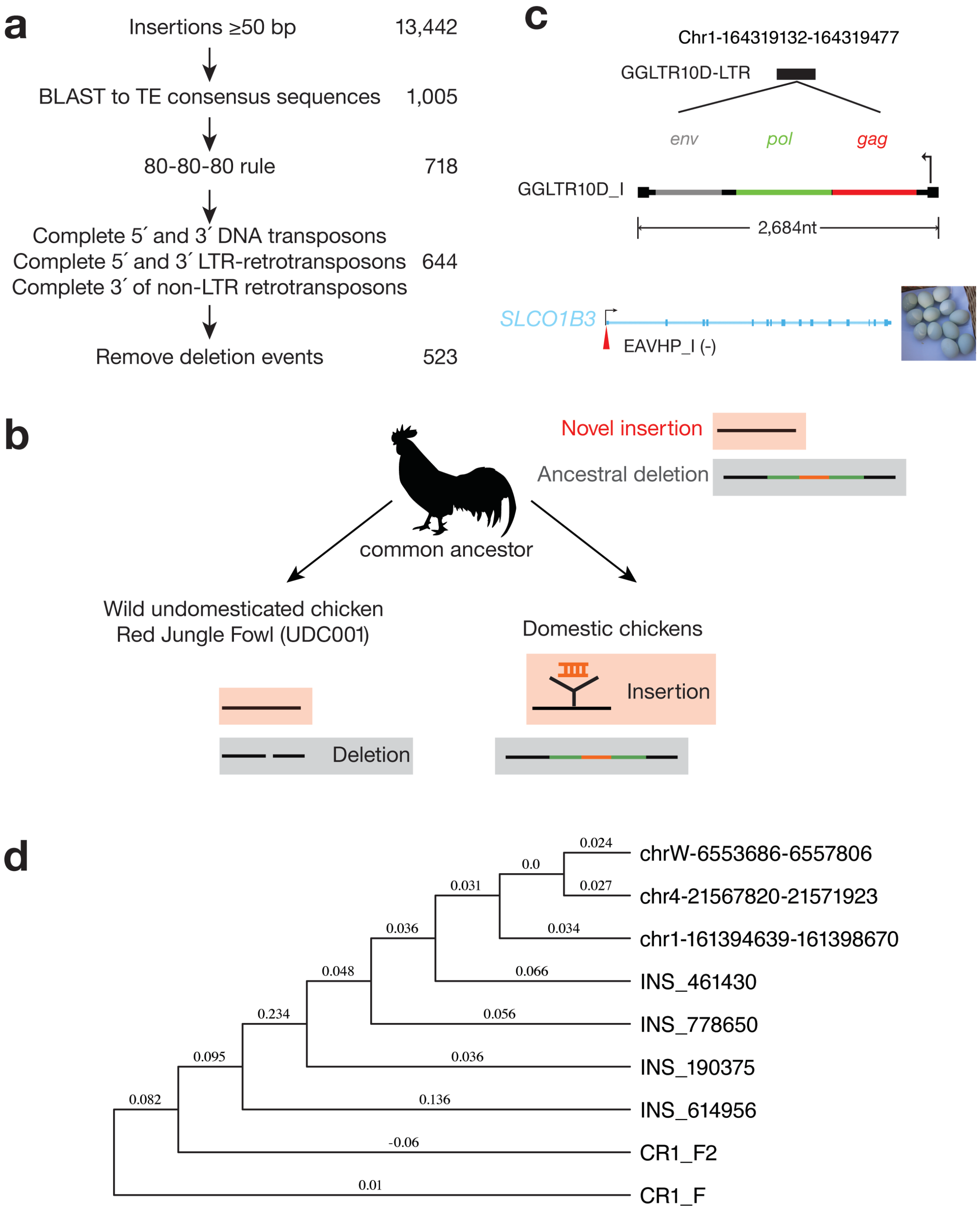

Supplementary Fig. 6, Related to Fig. 5. Pipeline to define active TE families in chickens. (a) Pipeline for identifying TE transpositions using ONT-sequencing reads. **(b)** The two evolutionary models, novel transposition (in pink) and ancestral deletion (in gray), explaining the insertions in domestic chickens using Red Jungle Fowl genome as reference. **(c)** (Top) Insertion of GGLTR10D_I into a solo LTR region (GGLTR10D-LTR), representing an ancestral deletion due to recombination. (Bottom) An example of transposition with EAVHP insertion at the promoter of *SLCO1B3* underlying the blue-shell egg traits in Araucana. Red triangle represents the insertion site. **(d)** Alignment of the four novel insertion sequences with the consensus sequences of CR1-F2 and CR1-F and three intact copies of CR1-F2 in the reference genome performed using MUSCLE program with default setting[1].

Supplementary Fig. 7.

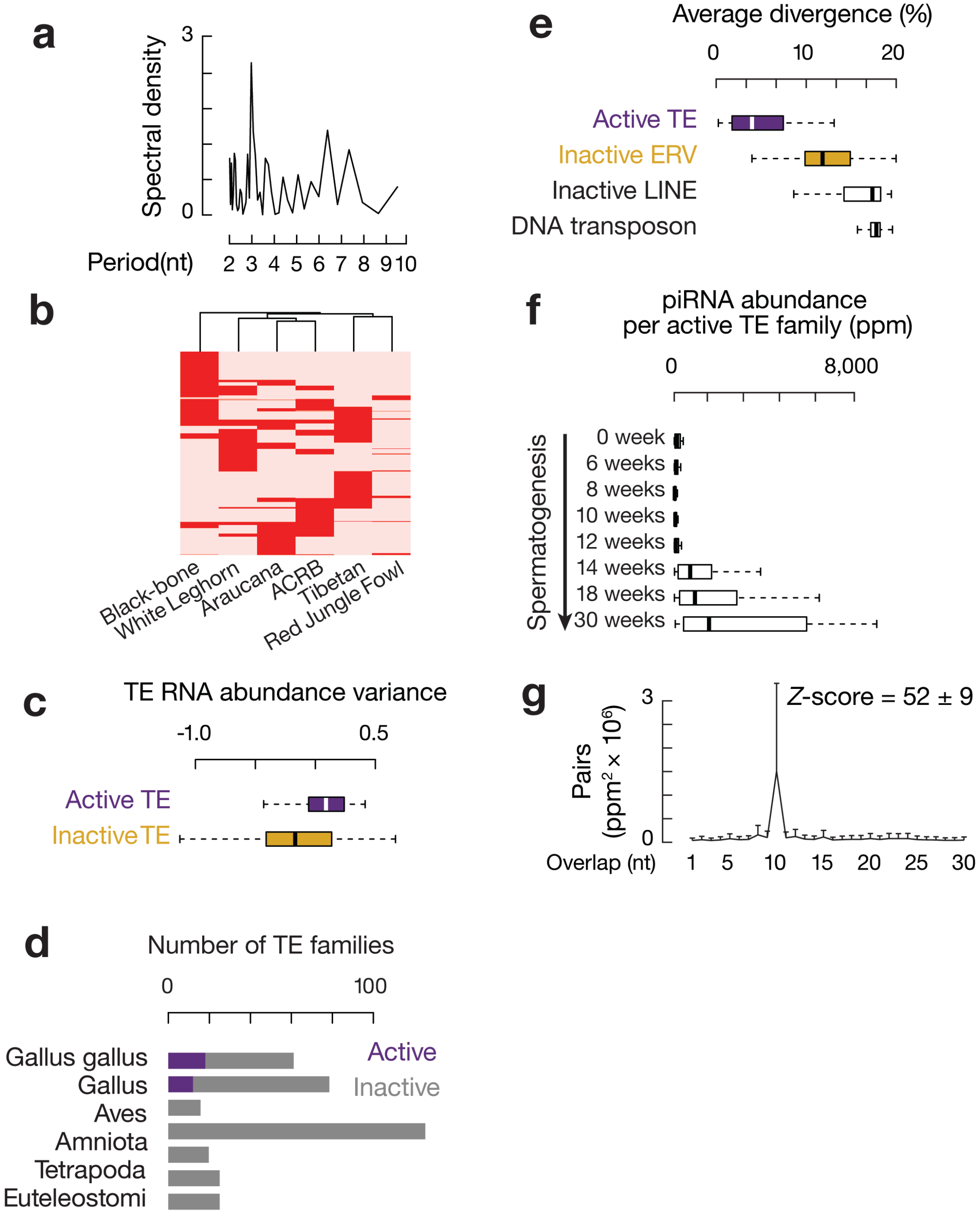

Supplementary Fig. 7, Related to Fig. 5. Conserved evolutionary force to suppress active TEs acts on pachytene piRNAs in chickens. (a) Discrete Fourier transformation of the distance spectrum of 5´-ends of ribosome protected fragments (RPFs) across active TEs in adult Red Jungle Fowl testes detected by Ribo-seq. **(b)** Heatmap of presence (red) or absence (pink) of each TE transposition in each chicken. **(c)** Box plots of the variance of RNA abundance per TE family among 19 chickens from 6 chicken breeds. **(d)** Histogram of TE ages. **(e)** Box plots of average divergence of TE insertions from their consensus sequences (Active TE n=30, Inactive ERV n=52, Inactive LINE n=63, DNA transposon n=100). Box plots show the 25th and 75th percentiles, whiskers represent the 5th and 95th percentiles, and midlines show median values. **(f)** Box plots showing piRNA abundance per TE family (n=245) as spermatogenesis progresses. Ppm, parts per million. Box plots show the 25th and 75th percentiles, whiskers represent the 5th and 95th percentiles, and midlines show median values. **(g)** The 5′-5′ overlap between sense and anti-sense piRNAs mapping to active TEs in adult chicken testes. The number of pairs of piRNA reads at each position is reported. Data are mean ± standard deviation (n=23).

Supplementary Fig. 8.

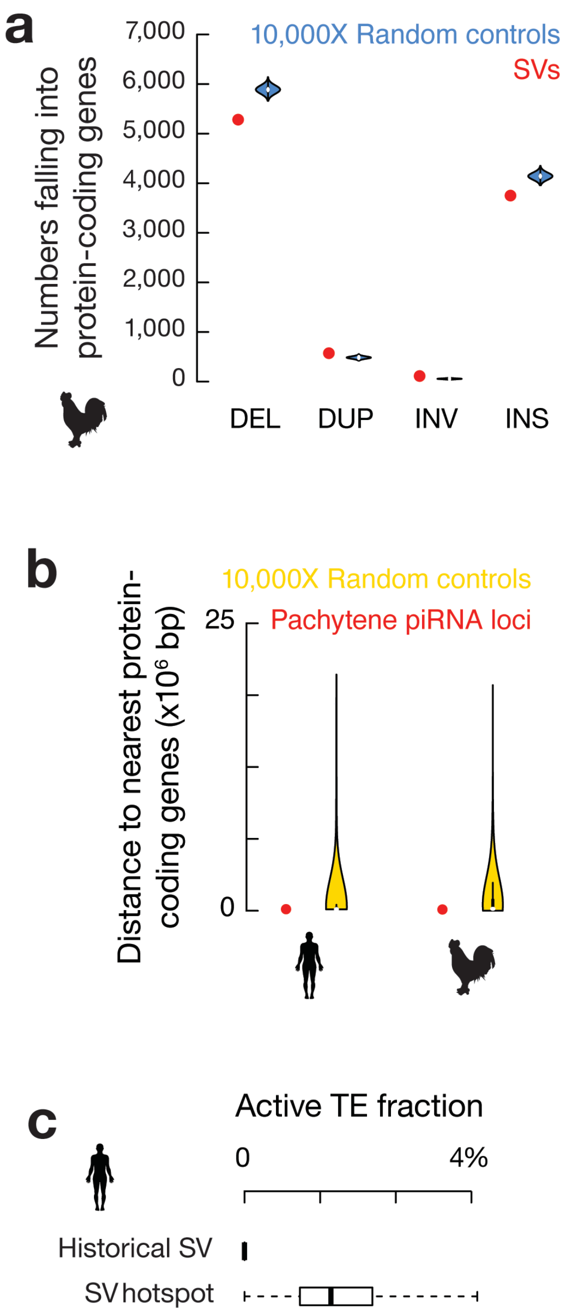

Supplementary Fig. 8. Pachytene piRNA loci are deleterious SV hotspots. (a) The number of SVs (red) and randomly shuffled control sequences (blue) falling into the protein coding regions (n=28,281). Violin plots represent the randomly shuffled control sequences that were computed 10,000 times. In comparison to random shuffle controls, deletions and insertions were significantly depleted from genic regions ($p < 1.0 \times 10^{-4}$) and duplications were slightly enriched at protein-coding regions ($p = 0.03$), while inversions were distributed randomly on chromosomes ($p = 0.63$). The majority of SVs are depleted from genic regions, a sign of negative selection, suggesting their deleterious impact on gene function. *p* value was calculated with one-tailed permutation test. **(b)** Median distance between protein coding genes and nearest pachytene piRNA loci (red), or nearest corresponding randomly shuffled control sequences (yellow). Violin plots represent the medians of randomly shuffled control sequences that were computed 10,000 times. **(c)** Boxplots represent the percentage of active TE sequences in historical SVs (n=17,789) and current SV hotspots (n=278) in humans. Box plots show the 25th and 75th percentiles, whiskers represent the 5th and 95th percentiles, and midlines show median values.

REFERENCES

1. Edgar, R. C. MUSCLE: a multiple sequence alignment method with reduced time and space complexity. *BMC Bioinformatics* **5**, 113 (2004).
