# Supplementary material for: Amniotes co-opt intrinsic genetic instability to protect germ-line genome integrity
Source: Nat Commun. 2023 Feb 13;14:812. doi: 10.1038/s41467-023-36354-x (PMC9925758; doi:10.1038/s41467-023-36354-x)
Supplement: Supplementary file 3 — Description of Additional Supplementary Files [file 41467_2023_36354_MOESM3_ESM.pdf]

Description of Additional Supplementary Files

File Name: Supplementary Data 1

Description: Detailed information and statistics for the sequencing data used in this study.

(A) Ribosome profiling sequencing statistics: reads and species.

(B) Small RNA sequencing statistics: reads and species.

(C) RNA-Seq statistics: reads and species.

(D) ONT-Seq statistics: reads and species.

(E) 245 TE families.

(F) 523 TE insertions.

(G) Genome coordinates for the 1,321 rooster piRNA-producing loci defined in this study are provided in UCSC BED format (i.e., 0-based) for galGal6.

(H) 189 tandem duplications in pachytene piRNA loci.

(I) 26 inversions in pachytene piRNA loci.

(J) 314 deletions in pachytene piRNA loci.

(K) 192 chicken SV hotspots.
