# Supplementary material for: Amniotes co-opt intrinsic genetic instability to protect germ-line genome integrity
Source: Nat Commun. 2023 Feb 13;14:812. doi: 10.1038/s41467-023-36354-x (PMC9925758; doi:10.1038/s41467-023-36354-x)
Supplement: Supplementary file 5 — Reporting Summary [file 41467_2023_36354_MOESM5_ESM.pdf]

nature portfolio

Corresponding author(s): Xin Zhiguo Li

Last updated by author(s): Jan 14, 2023

Reporting Summary

Nature Portfolio wishes to improve the reproducibility of the work that we publish. This form provides structure for consistency and transparency in reporting. For further information on Nature Portfolio policies, see our Editorial Policies and the Editorial Policy Checklist.

Statistics

For all statistical analyses, confirm that the following items are present in the figure legend, table legend, main text, or Methods section.

n/a	Confirmed	
☐	☒	The exact sample size (*n*) for each experimental group/condition, given as a discrete number and unit of measurement
☐	☒	A statement on whether measurements were taken from distinct samples or whether the same sample was measured repeatedly
☐	☒	The statistical test(s) used AND whether they are one- or two-sided<br>*Only common tests should be described solely by name; describe more complex techniques in the Methods section.*
☒	☐	A description of all covariates tested
☐	☒	A description of any assumptions or corrections, such as tests of normality and adjustment for multiple comparisons
☐	☒	A full description of the statistical parameters including central tendency (e.g. means) or other basic estimates (e.g. regression coefficient) AND variation (e.g. standard deviation) or associated estimates of uncertainty (e.g. confidence intervals)
☐	☒	For null hypothesis testing, the test statistic (e.g. *F*, *t*, *r*) with confidence intervals, effect sizes, degrees of freedom and *P* value noted<br>*Give P values as exact values whenever suitable.*
☒	☐	For Bayesian analysis, information on the choice of priors and Markov chain Monte Carlo settings
☒	☐	For hierarchical and complex designs, identification of the appropriate level for tests and full reporting of outcomes
☒	☐	Estimates of effect sizes (e.g. Cohen's *d*, Pearson's *r*), indicating how they were calculated

Our web collection on statistics for biologists contains articles on many of the points above.

Software and code

Policy information about availability of computer code

Data collection	RNAseq data were generated on HiSeq 2000 instrument (Illumina, San Diego, CA, USA). Oxford Nanopore Technologies data were generated on PromethION P48 (ONT-08-00443-02). Histology and immunostaining images were taken using Leica DM4000 B LED microscope system with Leica software: Leica Application Suite X (v1.1.0.12420).
Data analysis	General analyses of RNA sequencing, small RNA sequencing, Ribo-seq and degradome sequencing data were performed using piPipes pipeline (v1.4): https://github.com/bowhan/piPipes. Ribo-seq analysis used the pipeline we developed before: https://github.com/LiLabZhaohua/RiboSeqPipeline. The script for calculating spectral distribution from simulated negative controls is: https://github.com/LiLabZhaohua/LiLabScripts/blob/master/spectral_mid_100.py. For RNA-seq reads, the expression per transcript was normalized to the top quartile of expressed transcripts per library calculated by Cufflinks (v2.2.1), and the tpm (transcripts per million) value was quantified using Salmon (v0.8.2). Chicken transcriptome was assembled using StringTie (v1.3.3b) and the transcriptome annotation was performed using TransDecoder (v5.5.0), BlastP (v2.10.0+) and Hmmer (v3.3). Statistical analyses were performed in R (v3.5.0). Nucleotide periodicity was computed using GeneCycle (v1.1.4) package in R. ONT Sequencing data collected in this experiment were obtained as fast5 files, and after conversion of electric signals into base calls via the guppy (Oxford Nanopore Technologies, UK), the reads with mean qScore greater or equal to 7 were kept to continue subsequent bioinformatic analysis. The filtered data were aligned with the chicken genome (galGal6) using NGMLR (v0.27). The SVs were detected using Sniffles (v1.0.8). We also independently used SVIM (v1.4.2) to call SVs with default settings.

For manuscripts utilizing custom algorithms or software that are central to the research but not yet described in published literature, software must be made available to editors and reviewers. We strongly encourage code deposition in a community repository (e.g. GitHub). See the Nature Portfolio guidelines for submitting code & software for further information.

Data

Policy information about availability of data

All manuscripts must include a data availability statement. This statement should provide the following information, where applicable:

- Accession codes, unique identifiers, or web links for publicly available datasets
- A description of any restrictions on data availability
- For clinical datasets or third party data, please ensure that the statement adheres to our policy

Next-generation sequencing data used in this study have been deposited at the NCBI Gene Expression Omnibus under the accession number: GSE165330.

Human research participants

Policy information about studies involving human research participants and Sex and Gender in Research.

Reporting on sex and gender	NA
Population characteristics	NA
Recruitment	NA
Ethics oversight	NA

Note that full information on the approval of the study protocol must also be provided in the manuscript.

Field-specific reporting

Please select the one below that is the best fit for your research. If you are not sure, read the appropriate sections before making your selection.

☒ Life sciences ☐ Behavioural & social sciences ☐ Ecological, evolutionary & environmental sciences

For a reference copy of the document with all sections, see nature.com/documents/nr-reporting-summary-flat.pdf

Life sciences study design

All studies must disclose on these points even when the disclosure is negative.

Sample size	We either use at least three chickens from the same population to minimize individual variations, or combine > 22 chickens from diverse populations to include all the diversity in the chicken breeds. Our prior experience demonstrates that three replicates provide superb statistical power in sequencing analysis, allowing the elimination of false positive and negative changes in gene, RPF or piRNA expression.
Data exclusions	No data were excluded for analysis.
Replication	We include at least 3 biological replicates to compute our p value. All replicates were successful.
Randomization	NA
Blinding	Blinding is not relevant to our study as we include both positive and negative controls in each step of experiment procedure.

Reporting for specific materials, systems and methods

We require information from authors about some types of materials, experimental systems and methods used in many studies. Here, indicate whether each material, system or method listed is relevant to your study. If you are not sure if a list item applies to your research, read the appropriate section before selecting a response.

Materials & experimental systems

n/a	Involved in the study	
☐	☒	Antibodies
☒	☐	Eukaryotic cell lines
☒	☐	Palaeontology and archaeology
☐	☒	Animals and other organisms
☒	☐	Clinical data
☒	☐	Dual use research of concern

Methods

n/a	Involved in the study	
☒	☐	ChIP-seq
☒	☐	Flow cytometry
☒	☐	MRI-based neuroimaging

Antibodies

Antibodies used

Anti-CIWI antibody (1:100 dilution; Proteintech ,15659-1-AP);
Rabbit anti-SYCP1 antibody (1:100 dilution, Thermo Fisher, PA1-167630);
Rabbit anti-gamma-H2AX (1:250 dilution; Millipore, 05-636-1);
Secondary antibodies conjugated with Alexa Fluor 488 (Molecular Probes, Eugene, OR, USA)

Validation

All antibodies have been validated by the vendors or previous publications.

Animals and other research organisms

Policy information about studies involving animals; ARRIVE guidelines recommended for reporting animal research, and Sex and Gender in Research

Laboratory animals

The Athens Canadian Random Bred (ACRB) animals were raised under standard broiler and broiler breeder conditions under protocol (18083) approved by the International Animal Care and Use Committee (IACUC) at the University of Arkansas. White Leghorn Cornell Special C strain were raised and euthanized under the IACUC at Cornell University. Araucana were purchased from SkyBlueEgg Araucana (Winnfield, LA) and Awesome Araucana chicken hatchery (Redding, CA). Peking duck testes were purchased from a local farm (LeRoy, NY). Rooster testes from Red Jungle Fowl were collected from Hopkin Avian facility at UCD under the protocol #20591. The animal use protocol for sampling two indigenous village breeds, Tibetan chickens and Lvyang Blackbone chickens, were approved by the Animal Use and Care Committee of Northwest A&F University.

Wild animals

The study did not involve wild animals.

Reporting on sex

We used samples from roosters so only males were collected.

Field-collected samples

The study did not involve sample collected from the wild.

Ethics oversight

All experiments were reviewed and approved by the University of Rochester's University Committee on Animal Resources, performed in a PHS Assured and AAALAC, Int. accredited facility, and the study is compliant with all relevant ethical regulations regarding animal research.

Note that full information on the approval of the study protocol must also be provided in the manuscript.
