# Supplementary material for: Amniotes co-opt intrinsic genetic instability to protect germ-line genome integrity
Source: Nat Commun. 2023 Feb 13;14:812. doi: 10.1038/s41467-023-36354-x (PMC9925758; doi:10.1038/s41467-023-36354-x)
Supplement: Supplementary file 2 — Peer Review File [file 41467_2023_36354_MOESM2_ESM.pdf]

nature portfolio

Peer Review File

Amniotes co-opt intrinsic genetic instability to protect germ-line genome integrity

Open Access This file is licensed under a Creative Commons Attribution 4.0 International License, which permits use, sharing, adaptation, distribution and reproduction in any medium or format, as long as you give appropriate credit to the original author(s) and the source, provide a link to the Creative Commons license, and indicate if changes were made. In the cases where the authors are anonymous, such as is the case for the reports of anonymous peer reviewers, author attribution should be to 'Anonymous Referee' followed by a clear attribution to the source work. The images or other third party material in this file are included in the article's Creative Commons license, unless indicated otherwise in a credit line to the material. If material is not included in the article's Creative Commons license and your intended use is not permitted by statutory regulation or exceeds the permitted use, you will need to obtain permission directly from the copyright holder. To view a copy of this license, visit http://creativecommons.org/licenses/by/4.0/.

Reviewers' Comments:

Reviewer #1:
Remarks to the Author:
Mammalian pachytene piRNA genes diverge rapidly among mammals, even among modern humans. However, the extensive drifting rate of pachytene piRNA genes is result of neutral or positive selection remains mysterious. Sun et al has proposed a tempting model where evolution of pachytene piRNA genes in amniotes converge with that of structural variations (SVs) and such evolutionary force provides positive selection on the rapid divergence of pachytene piRNAs to suppress young and active transposons. Authors first annotated "pachytene piRNA genes" in several avian species; similar to mammals, avian pachytene piRNA genes evolve rapidly. They revealed that both mammalian and avian pachytene piRNA loci are enriched with SVs that further associates with the variation in pachytene piRNA sequences. Authors then examined the driving force that may possibly contribute to the emergence of SV hotspots in pachytene piRNA loci. The manuscript is very well-written and overall data support authors' conclusions. The findings contribute to our better understanding of how and why pachytene piRNA genes diverge rapidly.

1. Authors revealed that the majority of the pachytene piRNAs are expressed during transition from 12 to 18 weeks (line 82). What is the fraction of these piRNAs in total pachytene piRNA pool? Are those the ones diverge faster than the remaining?
2. In Extended Data S1C, it seems that CIWI also localizes to nuclei; what do authors think about it?
3. Do chicken pachytene piRNA genes not retain introns (Extended Data S2B)?
4. Figure 2A; iii: Authors should perform suitable statistics to claim piRNA loci have larger frequencies. Perhaps chi-square test p-value may help.
5. Line 148: I cannot follow how authors draw these conclusions (Fig 3a,b,c). What do authors mean by expression dosage? and compare to which clusters? how does cluster 542 overlap with inversion? Do authors claim that the piRNA dosage, relative abundance of different piRNA species from SV loci vary between chicken individuals? or the comparison is between loci with SV and loci without SV?
6. Judging from Extended Data S3D, there are ~20 human pachytene piRNA genes that retain SVs, are those the ones that are found in other eutherians? Authors should analyze SVs occupancies in human pachytene piRNA genes by their conservations: eutherian, primate-only, and human-specific. Incorporating this analysis will also be useful for the text on page 9 (lines 180-184), as authors should call the figure there.
7. Figure 4a: What does right violin plot represent? (for example in humans), is it all TE fraction?
8. What is the fraction of SVs in other long-noncoding RNA genes? Are SVs more represented within pachytene piRNA genes compared to lncRNA genes?
9. Authors reveal that pachytene piRNA genes are in fact not depleted of active TEs (a fraction of ~1.5% in mice) and that they silence active TEs. Authors should discuss the possible function of remaining pachytene piRNAs that diverge rapidly and are not derived from active TEs and SVs in discussion section.
10. Extended Data S2A: there is extra 'a' on the label residing at top of the left panel.

Reviewer #2:
Remarks to the Author:
The manuscript by Sun et al provides new data describing genomic origins of pachytene piRNAs in several non-model organisms. These results are a valuable resource for the small RNA field, enabling follow-up evolutionary studies. Yet several concerns need to be addressed before the manuscript is accepted for publication.

1. Authors' statement that pachytene piRNAs are required for TE silencing needs clarification. They likely mean that a subset of piRNAs present during meiosis is required for this function. But as was shown previously, these TE-silencing piRNAs do not originate from the conserved pachytene piRNA

producing loci, as transposons are not derepressed when these loci are removed (Wu et al., Nat Genet 2020, Choi et al., Plos Genet 2021). Mov10l1 CKO mice lack both types of such meiotic piRNAs (TE-targeting and non-TE piRNAs) and do not allow testing the function of each of those types separately, similar to CKO of Mili in Di Giacomo et al., Mol Cell 2013.

2. To make the manuscript easier to read, authors need to have all the necessary information either in the text or in figures. For example, p values are never accompanied with the information about what statistical test was used (including information whether it was one/two-tailed and whether adjustment for multiple comparisons was done). Most importantly, mentioning actual values in the text would help reader understand the effect size in comparisons: e.g., " … was higher (XXX vs. YYY; two-tailed ZZZ test, p = …)". Also, what was the Cre driving the CKO of Mov10l1?

3. Authors claim pachytene piRNA producing loci to be SV hotspots. To provide a more unbiased view of SV hotspots, it would be very useful to identify all genomic regions enriched for SVs and test for the overlap between these with piRNA loci. For example, the enrichment vs control is greater (~4-fold) for duplications and smaller (~1.5-fold) for deletions in Fig. 2C. The more global analyses of SV distribution would help put these enrichment differences in perspective. Besides, structural variants' nature and possible origins need to be described and discussed in more detail than it is currently done.

4. A minor comment: to avoid confusing CIWI with SIWI, I suggest authors use ChIWI instead. Also, please add that the protein name is, per nomenclature, PIWIL1.

Response to Reviewers' Comments

NCOMMS-22-28272

"**Amniotes co-opt intrinsic genetic instability to protect germ-line genome integrity**"

We thank the Reviewers for providing constructive feedback that has helped improve our manuscript. In this work, we used a comparative biology approach to investigate the function of pachytene piRNAs. Our study revealed a conserved function of pachytene piRNAs in defending active transposable elements (TEs), which efficiently rejected the possibility that pachytene piRNAs undergo neutral evolution. This function drives a convergent mechanism for piRNAs to keep up with the ever-changing TE repertoire across amniotes and explains why certain structural variations recur in the human population. We are pleased to see that both Reviewers recognize the importance of our findings and the quality of our research.

In this revision, we have adjusted the manuscript according to the reviewers' comments. First, we tested the overlap between SV hotspots and piRNA loci in the chicken genome (Extended Data Fig. 2f). Second, we tested the overlap between SVs and lncRNAs (Extended Data Fig. 2d) and revealed a different pattern compared to the overlap between SVs and piRNA loci (Fig. 2c). Third, we separated the piRNA loci according to their evolutionary age into *other eutheria*, *primates only*, or *human-specific* categories, and compared their overlap with piRNA loci, which further ruled out the "mutation" hypothesis (Extended Data Fig. 4a). Fourth, we expanded the mutational mechanisms section by ruling out the possibility that the hotspots are due to increased landing from active TE insertions (Extended Data Fig. 4f) and by moving the corresponding supplementary figures to a main figure (Fig. 4, with the previous Fig. 4 becoming Fig. 5). Fifth, we added a discussion on the potential function of non-TE piRNAs. Sixth, we have added detailed statistic tests on every *p-value* we have computed. We have highlighted these changes in the revision. Below, we provide a detailed point-by-point response to the Reviewers' concerns. We also hope the Reviewers appreciate the groundwork we provide here to move the study forward on pachytene piRNA evolution and function. We hope the Reviewers agree that the revised MS is now worthy of publication in *Nature Communications*.

Reviewer #1 (Remarks to the Author):

Mammalian pachytene piRNA genes diverge rapidly among mammals, even among modern humans. However, the extensive drifting rate of pachytene piRNA genes is result of neutral or positive selection remains mysterious. Sun et al has proposed a tempting model where evolution of pachytene piRNA genes in amniotes converge with that of structural variations (SVs) and such evolutionary force provides positive selection on the rapid divergence of pachytene piRNAs to suppress young and active transposons. Authors first annotated "pachytene piRNA genes" in several avian species; similar to mammals, avian pachytene piRNA genes evolve rapidly. They revealed that both mammalian and avian pachytene piRNA loci are enriched with SVs that further associates with the variation in pachytene piRNA sequences. Authors then examined the driving force that may possibly contribute to the emergence of SV hotspots in pachytene piRNA loci. The manuscript is very well-written and overall data support authors' conclusions. The

findings contribute to our better understanding of how and why pachytene piRNA genes diverge rapidly.

1. Authors revealed that the majority of the pachytene piRNAs are expressed during transition from 12 to 18 weeks (line 82). What is the fraction of these piRNAs in total pachytene piRNA pool? Are those the ones diverge faster than the remaining?

Response: In adult mammal testes, two groups of piRNAs can be detected based on their expression dynamics: pre-pachytene piRNAs and pachytene piRNAs. However, in chickens, as shown in Fig. 1c, the piRNAs we detected in adult testes, at least the ones produced from discrete genomic loci, are all pachytene piRNAs. This is further demonstrated by the lack of total piRNAs before the pachytene stage, as shown by Fig. 1a ii and Extended Data Fig. 1b ii. We have revised the manuscript to make this clearer. The lack of pre-pachytene piRNA in chickens suggests that pachytene piRNA is an ancestral form in amniotes, while pre-pachytene piRNA was acquired later during mammalian evolution. Considering this manuscript focused on pachytene piRNAs, we decided to discuss the origin of pre-pachytene piRNAs in a separate manuscript.

2. In Extended Data S1C, it seems that CIWI also localizes to nuclei; what do authors think about it?

Response: We thank the Reviewer for pointing this out. Nuclear function is indeed a key mystery for mammalian piRNAs. To test whether the nuclear signals of CIWI are valid, we are in the process of generating CIWI knockout chickens. We hope the Reviewer agrees that constructing a knockout chicken is beyond the scope of this manuscript.

3. Do chicken pachytene piRNA genes not retain introns (Extended Data S2B)?

Response: We thank the Reviewer for asking this question. Unfortunately, the current piRNA loci were defined computationally, so they are not transcriptional units. From the previous study on 3′ UTR piRNAs derived from mRNAs in chickens whose precursors contain introns[1], we would not be surprised if chicken piRNA precursors derived from lncRNA contain introns. We are in the process of analyzing single molecule long read sequencing on RNAs to define the precursor structures of chicken pachytene piRNA; however, we hope the Reviewer will agree that this is beyond the scope of the manuscript.

4. Figure 2A; iii: Authors should perform suitable statistics to claim piRNA loci have larger frequencies. Perhaps chi-square test p-value may help.

Response: We thank the Reviewer for pointing this out, and we apologize for the lack of clarity. We have performed a permutation test. In short, we generated a set of control regions by randomly shuffling SVs on the same chromosome and counted the number of

control regions falling into piRNA loci. We performed the above analysis 10,000 times to obtain a distribution of the frequency that occurs by chance and then compared the control distribution with the frequency of SVs at piRNA loci using the permutation test. We have revised the text to make this section clearer.

5. Line 148: I cannot follow how authors draw these conclusions (Fig 3a,b,c). What do authors mean by expression dosage? and compare to which clusters?

Response: We apologize for not making this analysis clear. Target recognition by small RNAs is impacted by the stoichiometry between small RNAs and target RNAs[2]. To test how SVs affect the abundance of extant piRNAs, we quantified piRNAs that uniquely map to all SV regions. Fig 3a is the representative figure to show the individual dosage variation. We quantified the variance of piRNA abundance per SV as described[3]. Basically, we calculated its median expression level and the coefficient of variation (CV) from the normalized read counts across individuals among 23 chickens from the 6 breeds previously mentioned. For the variance, we used the residuals from a locally weighted regression (LOESS) of the CV on median expression to obtain a measure of expression variation that is relative to the expected variation at a given expression level. The advantage of this method is that the expression variance is no longer correlated with the levels of expression[3]. This method was applied to calculate the variance for strand-bias and diversity. We found the duplicated regions displayed significantly higher abundance variance than that of piRNA loci lacking SVs ($p = 3.0 \times 10^{-8}$, Fig. 3d, left), suggesting that SVs in piRNA loci are associated with dosage changes of extant piRNAs.

how does cluster 542 overlap with inversion?

Response: We thank the Reviewers for asking this. Two piRNA loci overlap with the inversion, and two more flank the inversion. The two flanking loci served as control, as the strand bias was not affected. The two overlapping loci were mistakenly cut out during figure formatting. We apologize for the mistake and have fixed Fig. 3b.

Do authors claim that the piRNA dosage, relative abundance of different piRNA species from SV loci vary between chicken individuals? or the comparison is between loci with SV and loci without SV?

Response: The former is correct. The piRNA dosage, relative abundance of different piRNA species from SV loci, vary between chicken individuals significantly more than the loci without SVs. We have revised the text to make this clear.

6. Judging from Extended Data S3D, there are ~20 human pachytene piRNA genes that retain SVs, are those the ones that are found in other eutherians? Authors should analyze SVs occupancies in human pachytene piRNA genes by their conservations: eutherian, primate-only, and human-specific. Incorporating this analysis will also be useful for the text on page 9 (lines 180-184), as authors should call the figure there.

Response: We thank the Reviewer for this suggestion. We have performed the analysis suggested by the Reviewer and indeed the human-specific piRNA loci overlap significantly with the SV hotspots and with the pathogenic SVs. We have added the results as Extended Data Fig. 4a, and we have also added the discussion at the place recommended by the reviewer and where the human pathogenic SVs are analyzed.

7. Figure 4a: What does right violin plot represent? (for example in humans), is it all TE fraction?

Response: We apologize for not making the analysis clear. They were all LINE fractions. Since it has been well reported that all TEs are depleted from piRNA loci, we performed the analysis using LINEs as representatives. To be consistent with the flow of the text, we have redone the analysis using all TEs, including LINE, SINE, ERV, and DNA transposons, and the results have been updated in Fig. 5a (previously Fig. 4a).

8. What is the fraction of SVs in other long-noncoding RNA genes? Are SVs more represented within pachytene piRNA genes compared to lncRNA genes?

Response: We thank the Reviewer for the suggestions. We have performed a similar permutation test on lncRNAs as the test on piRNA loci shown in Fig. 2c. In the analysis, we used the lncRNA loci defined in this study based on the RNA-seq assembly as well as their coding potential. We have performed the proposed experiment, and now it is presented in Extended Data Fig. 2d. We did not see similar enrichment of SVs in the lncRNAs with only deletions enriched at the lncRNAs. While we do not quite understand why deletion is enriched, which might be related to certain types of lncRNAs, we hope you agree with us that it is beyond the scope of this manuscript.

9. Authors reveal that pachytene piRNA genes are in fact not depleted of active TEs (a fraction of ~1.5% in mice) and that they silence active TEs. Authors should discuss the possible function of remaining pachytene piRNAs that diverge rapidly and are not derived from active TEs and SVs in discussion section.

Response: We thank the Reviewer for pointing this out. It is indeed a fundamental question remaining in the piRNA field. We have added further discussions, and we believe the function of non-TE pachytene piRNAs is likely to facilitate the biogenesis of diverse pachytene piRNAs targeting active TEs. We have added the discussion as the following:

"We are also one step closer to understanding the function of the non-TE fraction of pachytene piRNAs. Because our studies have effectively rejected the possibility of pachytene piRNA loci undergoing neutral evolution, this raises three possible

explanations for the ubiquitous and abundant presence of non-TE piRNAs among amniotes. First, the production of non-TE piRNA is necessary for the biogenesis or function of piRNA targeting active TEs. Second, the non-TE pachytene piRNAs have their own sequence specific function. However, considering the stringent target recognition rules of piRNAs[4], the second possibility is unlikely to be true unless some auxiliary factors or unique subcellular environments loosen the pairing rules, which would further raise the challenge to distinguish self vs. non-self RNAs. Third, it is too expensive or difficult to eliminate these non-functional piRNAs. However, considering that the force to contract the genome is much stronger in avians, the extensive presence of non-TE piRNAs across amniotes is unlikely to be neutral. Further population genetics together with molecular biology is required to better distinguish these possibilities. "

10. Extended Data S2A: there is extra 'a' on the label residing at top of the left panel.

Response: Gratefully noted and fixed.

Reviewer #2 (Remarks to the Author):

The manuscript by Sun et al provides new data describing genomic origins of pachytene piRNAs in several non-model organisms. These results are a valuable resource for the small RNA field, enabling follow-up evolutionary studies. Yet several concerns need to be addressed before the manuscript is accepted for publication.

1. Authors' statement that pachytene piRNAs are required for TE silencing needs clarification. They likely mean that a subset of piRNAs present during meiosis is required for this function. But as was shown previously, these TE-silencing piRNAs do not originate from the conserved pachytene piRNA producing loci, as transposons are not derepressed when these loci are removed (Wu et al., Nat Genet 2020, Choi et al., Plos Genet 2021). Mov10l1 CKO mice lack both types of such meiotic piRNAs (TE-targeting and non-TE piRNAs) and do not allow testing the function of each of those types separately, similar to CKO of Mili in Di Giacomo et al., Mol Cell 2013.

Response: We thank the Reviewer for introducing the misconception in the field that TE-piRNAs are produced from prenatal or pre-pachytene stages but not pachytene piRNAs. The mentioned two studies[5,6] only removed one or two loci, and TE piRNAs are redundantly distributed among 100 pachytene piRNA loci. Therefore, TE are not derepressed[5,6]. *Mov10l1 CKO* mice driven by *Neurog3-cre* only block pachytene piRNAs but not earlier stages[7]. The *CKO* of *Mili*[8] is driven by *Stra8-Cre*, which express earlier than *Neurog3-cre*, and the spermatogenesis of *Mili CKO Stra8-Cre* mutants mostly arrest at pachytene stage. The lack of TE piRNAs produced before pachytene piRNAs triggers the pachytene checkpoint. We have unpublished results indicating that the *CKO* of *Mili* driven by *Neurog3-cre* arrests at the round spermatid stage, further supporting that the *Neurog3-cre* conditional knockout of piRNA biogenesis factor can distinguish early

piRNAs and pachytene piRNAs. Thus, the TE piRNAs expressed at the earlier stage alone are not sufficient to suppress TEs after pachytene stage. To further validate that the TE piRNAs are pachytene piRNAs, we have shown that these piRNAs are upregulated during pachytene stage (Extended Data Fig. 5b). We have revised the manuscript to clarify that the conditional mutants we used do not affect pre-pachytene piRNAs.

2. To make the manuscript easier to read, authors need to have all the necessary information either in the text or in figures. For example, p values are never accompanied with the information about what statistical test was used (including information whether it was one/two-tailed and whether adjustment for multiple comparisons was done). Most importantly, mentioning actual values in the text would help reader understand the effect size in comparisons: e.g., " ... was higher (XXX vs. YYY; two-tailed ZZZ test, p = ...)". Also, what was the Cre driving the CKO of Mov10l1?

Response: We thank the Reviewer for these suggestions. We have added the statistical test used to compute each p-value.

The Cre driving the CKO of Mov10l1 is *Neurog3-cre*[9,10]. We have added this information to the manuscript.

3. Authors claim pachytene piRNA producing loci to be SV hotspots. To provide a more unbiased view of SV hotspots, it would be very useful to identify all genomic regions enriched for SVs and test for the overlap between these with piRNA loci. For example, the enrichment vs control is greater (~4-fold) for duplications and smaller (~1.5-fold) for deletions in Fig. 2C. The more global analyses of SV distribution would help put these enrichment differences in perspective. Besides, structural variants' nature and possible origins need to be described and discussed in more detail than it is currently done.

Response: We thank the Reviewer for these suggestions. We defined the 192 SV hotspots of chickens and then tested their overlap with pachytene piRNAs. We defined SV hotspots following the previous analyses on human SVs[11], which did not separate the SV types (duplication, insertion, deletion, or inversion) but took them together as a whole. We found chicken SV hotspots significantly overlap with chicken pachytene piRNAs as shown in Extended Data Fig. 2d. Similar analyses have been performed between SV hotspots and pachytene piRNAs in humans reciprocally (Fig. 3e, middle, and Fig. S3d).

We expanded the section on the mutational mechanisms on SVs with the following changes: 1) we excluded the possibility that biased insertion of active TEs may contribute to hotspot formation at piRNA loci with the low number of novel TE insertions at piRNA loci and the considerable distance between novel TE integration sites (Extended Data Fig. 4f); and 2) we moved key results from the supplementary section as a new figure (Fig. 4).

4. A minor comment: to avoid confusing CIWI with SIWI, I suggest authors use ChIWI instead. Also, please add that the protein name is, per nomenclature, PIWIL1.

Response: We understand the validity of this comment. However, a number of publications have used the nomenclature CIWI[12–18], which we have been following. We are afraid that renaming will cause further inconsistency and consequently confuse the community. We can still pronounce it as ChIWI, as there are precedents such as "cello".

In summary, we believe our study presents a substantial advancement in the field as we uncover a conserved function of pachytene piRNA across amniotes, which drives the evolution of pachytene piRNAs and the distribution of SV hotspots in the genome. Our work suggests that recuring pathogenic SVs in the human population is the price paid to keep active TEs under control. While a complete understanding of the function of pachytene piRNAs requires extensive additional work, we believe our manuscript will stimulate new avenues of research aimed at answering these exciting questions.

1. Sun, Y. H. et al. Coupled protein synthesis and ribosome-guided piRNA processing on mRNAs. *bioRxiv* (2021).
2. Wu, P. H. & Zamore, P. D. Defining the functions of PIWI-interacting RNAs. *Nat Rev Mol Cell Biol* (2021).
3. Sigalova, O. M., Shaeiri, A., Forneris, M., Furlong, E. E. & Zaugg, J. B. Predictive features of gene expression variation reveal mechanistic link with differential expression. *Mol Syst Biol* **16**, e9539 (2020).
4. Arif, A. et al. GTSF1 accelerates target RNA cleavage by PIWI-clade Argonaute proteins. *Nature* **608**, 618-625 (2022).
5. Wu, P. H. et al. The evolutionarily conserved piRNA-producing locus pi6 is required for male mouse fertility. *Nat Genet* **52**, 728-739 (2020).
6. Choi, H., Wang, Z. & Dean, J. Sperm acrosome overgrowth and infertility in mice lacking chromosome 18 pachytene piRNA. *PLoS Genet* **17**, e1009485 (2021).
7. Zheng, K. & Wang, P. J. Blockade of pachytene piRNA biogenesis reveals a novel requirement for maintaining post-meiotic germline genome integrity. *PLoS Genet* **8**, e1003038 (2012).
8. Di Giacomo, M. et al. Multiple epigenetic mechanisms and the piRNA pathway enforce LINE1 silencing during adult spermatogenesis. *Mol Cell* **50**, 601-608 (2013).
9. Schonhoff, S. E., Giel-Moloney, M. & Leiter, A. B. Neurogenin 3-expressing progenitor cells in the gastrointestinal tract differentiate into both endocrine and non-endocrine cell types. *Dev Biol* **270**, 443-454 (2004).
10. Sun, Y. H. et al. Ribosomes guide pachytene piRNA formation on long intergenic piRNA precursors. *Nat Cell Biol* **22**, 200-212 (2020).
11. Ebert, P. et al. Haplotype-resolved diverse human genomes and integrated analysis of structural variation. *Science* (2021).
12. Kim, T. H. et al. Conserved functional characteristics of the PIWI family members in chicken germ cell lineage. *Theriogenology* **78**, 1948-1959 (2012).

13. Rengaraj, D. et al. Small non-coding RNA profiling and the role of piRNA pathway genes in the protection of chicken primordial germ cells. *BMC Genomics* **15**, 757 (2014).
14. Sohn, Y. A. et al. The CCAAT element in the CIWI promoter regulates transcriptional initiation in chicken primordial germ cells. *Mol Reprod Dev* **81**, 871-882 (2014).
15. Rybecká, S., Štítkovcová, K., Vychytilová-Faltejsková, P. & Slabý, O. Zapojení PIWI-interagujících RNA do procesů kancerogeneze prostřednictvím regulace genové exprese. *Klinická onkologie* 428 (2016).
16. Sun, Y. H. et al. Domestic chickens activate a piRNA defense against avian leukosis virus. *Elife* **6**, (2017).
17. Chang, K. W. et al. Stage-dependent piRNAs in chicken implicated roles in modulating male germ cell development. *BMC Genomics* **19**, 425 (2018).
18. Li, X. Z. What can PIWI-interacting RNA research learn from chickens, and vice versa. *Canadian Journal of Animal Science* **99**, 641-648 (2019).

Reviewers' Comments:

Reviewer #1:
Remarks to the Author:
Authors addressed all my concerns; manuscript should be accepted for publication.

Reviewer #2:
Remarks to the Author:
The authors have addressed my concerns. I recommend publishing the revised version.
